# Cross-kingdom noncoding RNA regulation facilitates *Nosema bombycis* proliferation

**DOI:** 10.1016/j.engmic.2026.100278

**Published:** 2026-06-03

**Authors:** Pengcheng Zhang, Boyuan Deng, Wenxuan Fang, Feifei Liu, Peng Chen, Xuhua Huang, Minhui Pan, Zhanqi Dong

**Affiliations:** a State Key Laboratory of Resource Insects, Southwest University, Chongqing 400716, China; b Key Laboratory of Sericultural Biology and Genetic Breeding, Ministry of Agriculture and Rural Affairs, Southwest University, Chongqing 400716, China; c Guangxi Key Laboratory of Sericultural Genetic Improvement and Efficient Breeding, Guangxi Research Academy of Sericultural Science, Nanning, Guangxi Zhuang Autonomous Region, Nanning 530007, China

**Keywords:** *Bombyx mori*, *Nosema bombycis*, *NbLNC2914*, Bmo-miR-2808a-3p, *NBO_58g0005*

## Abstract

Microsporidia are obligate intracellular fungal parasites that are widely distributed in natural and agricultural environments and represent an important biological hazard to economically important insects. *Nosema bombycis* (*N. bombycis*)*,* the causative agent of pébrine disease, poses a serious threat to silkworm breeding and sericultural sustainability. However, the molecular mechanisms underlying microsporidian proliferation and host-pathogen interactions remain poorly understood. In this study, we systematically characterized the long noncoding RNAs (lncRNAs) and microRNA-like RNAs (milRNAs) of *N. bombycis*, an important pathogen of *Bombyx mori* (*B. mori*). Using the full-length transcriptome and small RNA sequencing data from *B. mori* midgut tissues infected with *N. bombycis* at key developmental stages, we identified 30 novel lncRNAs and 12 previously unreported milRNAs in *N. bombycis*. We further established a competing endogenous RNA regulatory network involving *N. bombycis*-derived lncRNAs, parasite-specific milRNAs, and host-derived microRNAs (miRNAs), revealing intricate molecular interactions that may be implicated in pathogenicity. Notably, *NbLNC2914*, a highly expressed lncRNA, was shown to function as a molecular sponge for the host miRNA bmo-miR-2808a-3p, modulating its expression and consequently regulating the downstream *N. bombycis* gene, *NBO_58g0005*. Functional validation assays demonstrated that this regulatory axis had an important impact on *N. bombycis* proliferation. Our findings reveal a previously unrecognized noncoding RNA (ncRNA)-mediated regulatory axis that facilitates *N. bombycis* proliferation within host cells. This study provides mechanistic insights into the molecular basis of microsporidia pathogenicity and highlights ncRNAs as potential molecular targets for mitigating microsporidia-associated biological hazards in agricultural ecosystems.

## Highlights

• 30 novel lncRNAs and 12 novel milRNAs were identified in *N. bombycis*.
• A ceRNA network of *N. bombycis* lncRNAs, milRNA, and host miRNAs was constructed.
• NbLNC2914 as a bmo-miR-2808a-3p sponge to regulate parasite proliferation.
• The bmo-miR-2808a-3p suppressed the expression of *NBO_58g0005* gene.

## Graphical abstract

**Figure:**
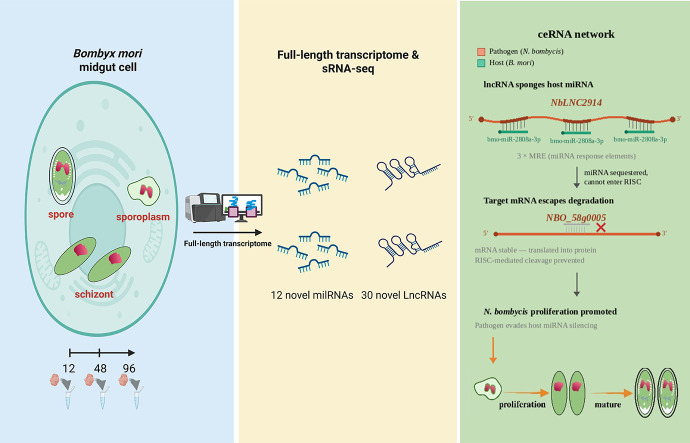

## 1 Introduction

Microsporidia are unicellular eukaryotes that are obligate intracellular parasites. These organisms have the smallest genomes of any eukaryote, ranging from 2.3 to 24 Mbp [[1], [2], [3]]. The microsporidian genome is characterized by the loss of many genes and an extremely compact structure [4]. During the long parasitic evolution of microsporidia, these organisms have lost many genes and biochemical pathways and are now only able to obtain energy through glycolysis, although they can still obtain energy from the host [5]. This massive loss of genes is only one reason for microsporidian genome reduction, which has resulted in considerable shortening of genes and intergenic regions [6]. In extreme cases, the average length of the intergenic region of *Encephalitozoon intestinalis* (*E. intestinalis*) is only about 100 bp, and several protein-coding regions overlap [7]. Analysis of the genomic differences between *E. intestinalis* and *Encephalitozoon cuniculi* (*E. cuniculi*) has shown that the noncoding regions of the genome are highly conserved and evolved at a slower rate than the protein-coding regions. This suggests that the intergenic regions retained by microsporidia under selective pressure may have functional importance for organismal survival [8]. The genome size of *Nosema bombycis* (15.7 Mbp) was substantially larger than that of *Nosema antheraeae* (6.6 Mbp) and *Nosema ceranae* (7.9 Mbp). In addition, the gene density of *N. bombycis* (0.34 gene/kbp) was shown to be comparable to that of *N. ceranae* (0.33 gene/kbp), but lower than that of *N. antheraeae* (0.53 gene/kbp), suggesting that *N. bombycis* contains more redundant sequences and noncoding regions [9]. However, the production of noncoding RNAs and their biological functions in conserved noncoding regions remain unclear.

Noncoding RNAs (ncRNAs) are a class of RNA molecules that are not translated into proteins and participate in a wide range of biological functions, including transcription, translation, and epigenetic modifications [10]. ncRNAs are classified into two types based on their size: small ncRNAs (17–200 nucleotides [nt]) and long ncRNAs (>200 nt). Small RNAs, including microRNAs (miRNAs), small interfering RNAs (siRNAs), and PIWI-interacting RNAs (piRNAs), typically function as negative regulators by interfering with the expression of target RNAs [11,12]. Conversely, long noncoding RNAs (lncRNAs) are a diverse group of transcripts whose functions are not fully understood [13]. In recent years, increasing evidence has shown that ncRNAs play important regulatory roles in fungal species. Fungal microRNA-like RNAs (milRNAs), first identified in *Neurospora*, have expanded the repertoire of fungal regulatory small RNAs and highlighted the importance of RNA interference-related pathways in fungi [14,15]. Transcriptomic studies have revealed that fungi encode abundant lncRNAs, many of which are associated with development, stress adaptation, metabolism, and pathogenicity [[16], [17], [18]]. These findings suggest that ncRNA-based regulation is an important component of fungal biology and provide a conceptual basis for investigating the ncRNA-mediated regulation of microsporidia. Huang et al. [19,20] predicted and validated nine milRNAs in *N. ceranae* using full-length transcriptome sequencing. Subsequent studies have identified various types of ncRNAs in microsporidia, including snoRNAs, snRNAs, lncRNAs, and circRNAs, but these studies have primarily focused on predicting the characteristics and functions of these ncRNAs [21]. We previously used full-length transcriptome sequencing to screen and validate 11 milRNAs in *N. bombycis*-infected cell samples. *N. bombycis* milRNA-8 is capable of repressing the expression of *B. mori* peroxisomal membrane protein gene 16 (*BmPEX16*) across kingdoms, thereby inhibiting the host peroxisomal metabolic pathway and increasing its susceptibility to *N. bombycis* infection [22]. However, *N. bombycis* ncRNAs have not been thoroughly studied, and it remains unclear whether ncRNAs are involved in the regulation of their host interactions.

The life cycle of microsporidia typically begins when the host ingests infectious spores. Upon entering the host cell, the spore germinates, releasing the infective sporoplasm, which then infects the host cells [23]. Once inside the host cell, microsporidia proliferate via merogony (asexual replication) before undergoing sporogony, where new spores are formed. These newly formed spores are subsequently released from the host cell, after which they infect new cells and continue their cycle [24]. This life cycle allows microsporidia to survive and thrive in harsh intracellular environments, often resulting in chronic infections [25]. In this study, we sampled infected *Bombyx mori* midguts at three distinct time points post-infection: 12, 48, and 96 h. The 12-h time point corresponds to the early phase of spore germination, during which the microsporidia begin to actively infect and replicate within the host. The 48-h time point represents the schizogony phase, when the parasites undergo rapid replication, leading to significant parasite proliferation. Finally, the 96-h time point captures the spore maturation phase, in which the microsporidia complete their asexual reproduction, forming mature spores ready to exit the host and continue the cycle. These time points were selected to capture the key stages of microsporidian development and enable detailed analysis of gene expression dynamics during infection. In this study, we identified *N. bombycis* ncRNAs by full-length transcriptome sequencing and constructed a ceRNA-based regulatory network of lncRNAs. We also identified the function of an lncRNA, suggesting that lncRNAs play an important role in microsporidia development. The lncRNA *NbLNC2914* was functionally analyzed and found to target bmo-miR-2808a-3p, thereby regulating the expression of the downstream gene *NBO_58g0005*. The results of this study provide further insights into the function of ncRNAs in microsporidia and support the role of ncRNAs in the biological processes of pathogen-host interactions.

## 2 Materials and methods

### 2.1 Materials and reagents

BmE-SWU1 cells were maintained in our laboratory and cultured at 27 °C in Grace medium (United States Biological, USA) containing 10% (v/v) fetal bovine serum (BioAgrio, USA) with penicillin–streptomycin (Thermo Fisher, USA) [26]. *B. mori* larvae of the Dazao strain were reared in our laboratory under standard conditions. *N. bombycis* strain CQ1 (CVCC No 102,059) was obtained from the China Veterinary Culture Collection Center [27]. An anti-Nb tubulin polyclonal antibody for fluorescence in situ hybridization (FISH) was prepared in our laboratory. The *NbLNC2914* FISH probe consisted of a mix of oligonucleotide probes labeled with digoxin, including a bmo-miR-2808a-3p FISH probe labeled with biotin, avidin-Red, anti-Digoxin horseradish peroxidase -conjugate, and TSA-488 purchased from Exonbio (Guangzhou, China). Alexa Fluor 555-conjugated goat anti-rabbit secondary antibody was purchased from Thermo Fisher Scientific (USA).

### 2.2 *N. bombycis* collection

Fourth-instar silkworm larvae were orally fed a suspension containing 10^5 *N. bombycis* spores per larva using a micropipette. When the silkworm larvae had grown to the pupal stage (6 days), the midgut was discarded, the pupal contents were collected, and the mixture was filtered through cotton three times. The filtrate was centrifuged three times at 500 × g for 5 min, and the resulting supernatant was collected. The remaining precipitate was resuspended in buffer and further centrifuged three times at 3000 × g for 5 min. The spores were collected from the final precipitate and enumerated based on the average count after each sample was counted three times using a hemocytometer.

### 2.3 Library construction and data analysis

Total RNA was extracted from the control (12, 48, and 96 h post-infection) and *N. bombycis* infection group (12, 48, and 96 h p.i.) using AG RNAex Pro reagent according to the manufacturer’s instructions (Agbio, China). Allwegene (Beijing, China) conducted both the library construction and Illumina sequencing. A total of 3 μg of RNA per sample was used as input material for the RNA sample preparations. For lncRNA library preparation, messenger RNA (mRNA) was first enriched by removing rRNA using the Epicentre Ribo-zero™ rRNA Removal Kit (Epicentre, USA), resulting in rRNA-depleted RNA samples. These samples were then used for library construction with the NEBNext Ultra™ RNA Library Prep Kit (NEB, USA) according to the manufacturer’s protocol. During library preparation, the cDNA fragments were purified using the AMPure XP system (Beckman Coulter, Beverly, USA) to select fragments of 150–200 bp in length. Finally, PCR amplification was performed to construct cDNA libraries. For small RNA library preparation, sequencing libraries were generated using a NEBNext® Multiplex Small RNA Library Prep Set for Illumina® (NEB, USA) following the manufacturer’s recommendations, with index codes added to attribute sequences to each sample. Following PCR amplification, the target DNA fragments were separated by polyacrylamide gel electrophoresis (PAGE), after which the cDNA library was obtained by gel excision and purification. The quality of the library was assessed using an Agilent Bioanalyzer 2100 system with DNA High Sensitivity Chips. The libraries were pooled according to their effective concentration and the desired data volume. Finally, an Illumina NovaSeq6000, PE150 sequencing strategy was used. Raw sequence reads are available in the NCBI database under accession No PRJNA1273716.

### 2.4 Identification of lncRNAs and milRNAs

TopHat v2.0.9 [28] was used for reference genome alignment analysis of the filtered sequences. Four methods, namely CPC analysis [29], CNCI analysis [30], CPAT analysis [31], and pfam protein structural domain analysis [32], were used to identify lncRNAs, and the intersection of the four results was selected as candidate lncRNAs. Small RNA tags were mapped to the *N. bombycis* reference genome by Bowtie [33] without mismatch, after which the mapped small RNA tags were employed to search for known miRNAs using miRBase22.0 as a reference.

### 2.5 Total RNA extraction and expression analysis

Total RNAs of various samples from mature spores, *N. bombycis*-infected BmE cells, and the *N. bombycis*-infected midgut were extracted using TRIzol reagent (Agbio, China) according to the manufacturer’s protocols. Next, the total RNAs were used for cDNA synthesis with the PrimeScript™ RT reagent Kit (Takara, Japan) and miRNA first-strand cDNA synthesis (Tailing Reaction) (Sangon Biotech, China). The cDNAs were then used as templates for qPCR with amplifications performed as previously described (miR6498). Subsequently, data were analyzed based on the 2^^–ΔΔCt^ method using NbsnRNA U6 and Nbα-Tubulin as endogenous controls to quantify the expression levels of milRNA and mRNA, respectively. The primers used are listed in Supplementary Table S3.

### 2.6 Immunofluorescence–Fluorescence in situ hybridization

FISH reagents were formulated using DEPC water. The FISH assay was performed using a D-T-G type miRNA in situ hybrid kit (Exonbio, China) according to the manufacturer’s protocol. The dilutions of the antibodies and probes used were as follows: *NbLNC2914* Probe:lncRNA hybridization buffer, 1:200; miRNA probe:miRNA hybridization buffer, 1:200; anti-digoxin HRP conjugate:α-NbTubulin rabbit polyclonal antibody:blocking buffer, 1:1:398; anti-digoxin HRP conjugate:avidin-Red:blocking buffer, 1:1:398; Alexa-fluor 555:100 × DAPI:blocking buffer, 1:5:494. All samples were observed under a super-resolution laser confocal microscope (FV3000; Olympus Corporation, Tokyo, Japan) with appropriate channels.

### 2.7 Construction of expression plasmids

The 5′ and 3′ ends of the target lncRNA were obtained via Rapid Amplification of cDNA Ends (RACE) PCR with a SMARTer® RACE 5′/3′ Kit (Takara, Dalian, China) following the manufacturer’s protocol. The complete sequence of *NbLNC2914* was cloned into the pIZ/V5-His plasmid and named pIZ-*NbLNC2914*. The *B. mori* miR-2808a-3p expression frame, which includes the U6 promoter, miR-2808a-3p mature sequence, and TTTTTT termination signal, was cloned into the Puro-mCherry vector and named Puro-miR-2808a-3p The clonal primers and other siRNA sequences are listed in Table S3.

### 2.8 Dual-luciferase reporter assays

The complete sequence of *NbLNC2914* was cloned into the 3′UTR dual-luciferase expression plasmids of firefly luciferase (pGL3-IE1-Fluc) and denoted pGL3-WT-*NbLNC2914*-Fluc. The binding sites of *NbLNC2914* with miR-2808a-3p were predicted and found to be partially mutated. The mutant *NbLNC2914* was subsequently cloned into pGL3-IE1-Fluc and named pGL3-MUT-*NbLNC2914*-Fluc. The FLuc reporter vector, pGL3-IE1-Rluc (with Rluc as an internal reference gene), and milRNA-related vectors were co-transfected into BmE-SWU1 cells at a molecular ratio of 4:1:4. At 24–48-h post-transfection (hpi), the cells were collected and luciferase activity was detected using a Dual-Luciferase Reporter Gene Assay Kit (Yeason, China). Finally, the relative luciferase activity (Fluc/Rluc) was normalized to the values obtained using pGL3-Basic as the control plasmid.

### 2.9 *N. bombycis* protein extraction

The 10^9^ microsporidia were transferred into centrifuge tubes, after which 200 μg of 425–600-μm acid-washed glass beads (Merck, USA) were added. The tubes were subsequently placed in a BeadBeater and shaken at 7000 × g for 20 s, nine times. After bead beating, 300 μL of cell lysis buffer, PMSF, and RNase inhibitor (Beyotime, China) were added to the centrifuge tubes, and the mixture was incubated at 4 °C for 6 h. Finally, the samples were centrifuged at 13,400 × g for 10 min, before the supernatant was collected and stored at –80 °C.

### 2.10 RNA pulldown

The *NbLNC2914*-binding proteins were examined using RNA pull-down assays conducted with a Pierce Magnetic RNA-Protein Pull-Down Kit (Thermo Fisher Scientific) according to the manufacturer’s instructions. Biotinylated *NbLNC2914* and antisense sequences were synthesized using a T7 High-Yield RNA Synthesis Kit (Yeason, China). The *N. bombycis* total proteins were incubated overnight with biotinylated *NbLNC2914*, and then precipitated with streptavidin magnetic beads. Finally, the retrieved protein was eluted from the RNA-protein complex, analyzed by immunoblotting or silver staining, and subjected to liquid chromatography-mass spectrometry analysis with a Dionex Ultimate 3000 nano-LC system and Thermo Fisher Q-Exactive (Thermo Fisher, USA).

### 2.11 Statistical analyses

Each experiment was repeated at least three times, and the values are presented as the means ± standard deviations (SD). Mean values between pairs of treatments were compared using Student’s *t*-tests. Statistical significance was set at a *P*-value < 0.01. All statistical analyses were conducted using GraphPad Prism 7 software (https://www.graphpad.com).

## 3 Results

### 3.1 *N. bombycis* encodes numerous long ncRNAs and miRNA-like RNAs

**Fig. 1:**
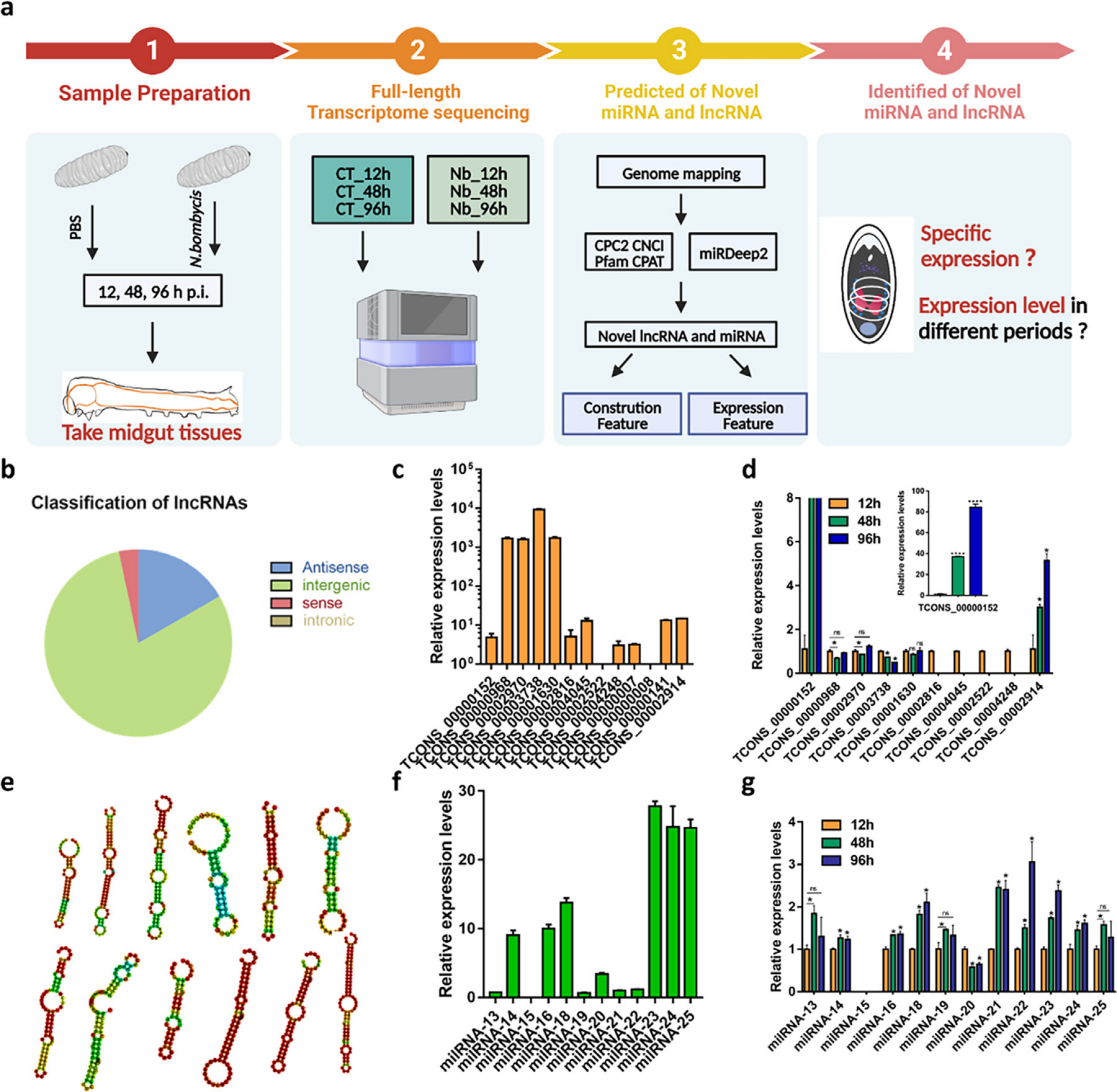
Identification of novel lncRNAs and milRNAs in *N. bombycis*.

To investigate the expression of ncRNAs in *N. bombycis*, we initially examined the characteristics of *N. bombycis* proliferation in the midgut of infected silkworms. Our results showed that *N. bombycis* was in the spore germination phase at 12 hpi, the schizogony phase at 48 hpi, and the spore maturation phase at 96 hpi (Fig. S1). We then collected midgut samples from infected silkworms at 12, 48, and 96 hpi (NB_12h, NB_48h, and NB_96h) and uninfected silkworm midgut samples at the same time points (CT_12h, CT_48h, CT_96h) for full-length transcriptome sequencing (Fig. 1a). We obtained 7.0 Gb of clean lncRNA data and 2.5 Gb of clean sRNA data, with Q20 and Q30 values greater than 80%, indicating high-quality raw data (Table S1). Next, we predicted novel lncRNAs using four methods: CPC, CNCI, CPAT, and pfam protein structural domain analysis, while new milRNAs were predicted using miREvo and miRDeep2miRNA. We also analyzed sequencing data from previously analyzed *N. bombycis*-infected cells. Thirty lncRNAs and 12 miRNAs were predicted (Fig. S2 and Table S2).

(a) Diagram showing the process of identifying *N. bombycis* lncRNAs and miRNAs. (b) Classification of identified novel_lncRNAs. (c) Identification of *N. bombycis*-specific expression of lncRNAs. (d) qRT-PCR validation of differential lncRNAs expression levels. (e) Predicted secondary structures of novel milRNAs. From left to right, the upper row shows milRNA-13, milRNA-14, milRNA-15, milRNA-16, milRNA-18, and milRNA-19, and the lower row shows milRNA-20 to milRNA-25. (f) Identification of microsporidia-specific expression of milRNAs. (g) qRT-PCR validation of differential milRNAs expression levels.

Of the 30 predicted lncRNAs, 24 were long intergenic noncoding RNAs (lincRNAs), five were antisense lncRNAs, and one was a sense lncRNA; however, no intronic lncRNAs were identified (Fig. 1b). To confirm that these lncRNAs were specifically expressed by *N. bombycis* and to evaluate changes in their expression levels, we selected the top nine lncRNAs expressed in midgut samples and the four lncRNAs identified in cell samples and analyzed their expression in mature spores, as well as in samples collected at different time points after midgut infection. We found that each lncRNA was expressed in *N. bombycis* (Fig. 1c), indicating that the lncRNAs identified using high-throughput sequencing were specifically expressed in *N. bombycis*. Furthermore, the expression of TCONS_00000152 and TCONS_00002914 gradually increased with *N. bombycis* proliferation, whereas TCONS_00003738, TCONS_00002816, TCONS_00004045, TCONS_00002522, and TCONS_00004248 were highly expressed at 12 hpi but were not expressed or expressed at lower levels at subsequent time points. TCONS_00000968 and TCONS_00002970 showed lower expression levels at 48 hpi, whereas TCONS_00001630 showed no change in expression levels over time (Fig. 1d). These distinct expression patterns suggest that lncRNAs are involved in various biological processes.

To evaluate the newly identified milRNAs, we first conducted secondary structure predictions, which revealed that 12 had typical secondary structures (Fig. 1e). To confirm that milRNAs were specifically expressed by *N. bombycis* and to evaluate changes in their expression levels, we measured the expression levels of novel milRNAs in mature spore samples and samples from different time points after midgut infection using poly(A)-tailing reverse transcription followed by quantitative PCR (qRT-PCR). We found that, except for milRNA-15, all milRNAs were expressed in mature spore samples, demonstrating that they are specifically expressed in *N. bombycis* (Fig. 1f). Following infection, the expression levels of most of the new milRNAs gradually increased with *N. bombycis* development. milRNA-13, milRNA-14, milRNA-16, and milRNA-21 were highly expressed at 48 hpi, while milRNA-20 was highly expressed at the spore germination stage and milRNA-15 was not present at any time point (Fig. 1g).

### 3.2 Construction of the ceRNA network of *N. bombycis*

**Fig. 2:**
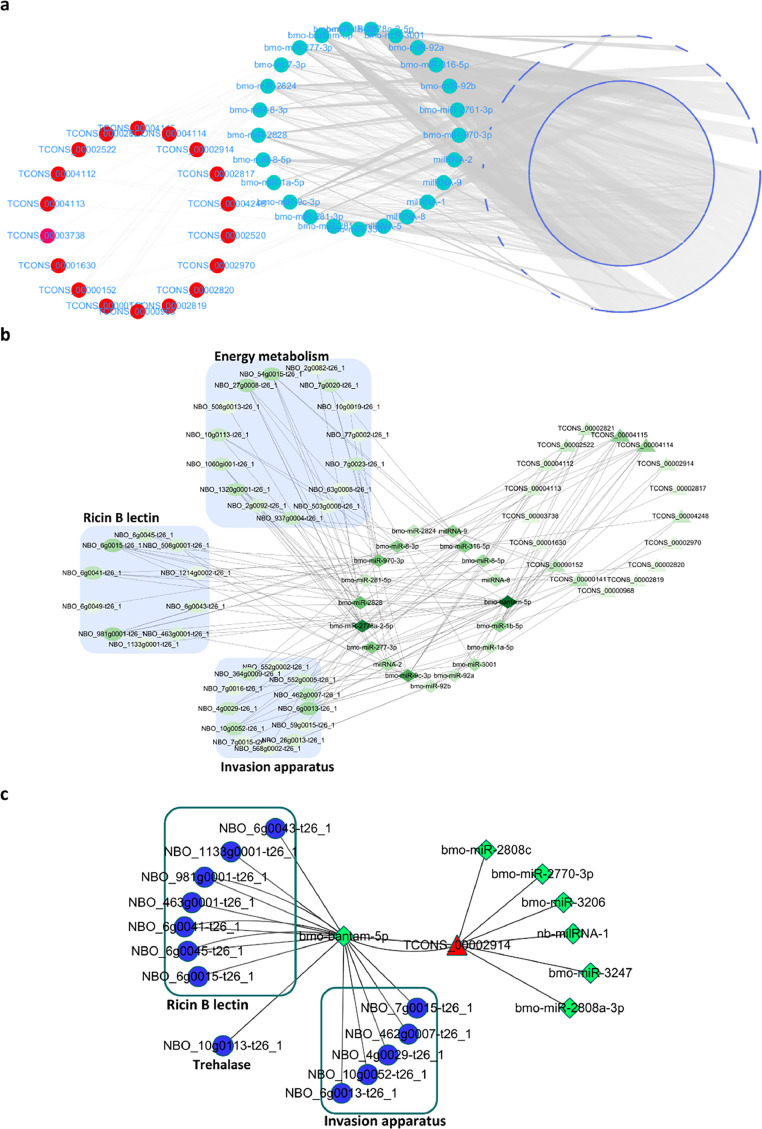
Construction of the ceRNA network of *N. bombycis*.

One mechanism by which lncRNAs function is by acting as molecular sponges to regulate the expression of miRNAs. To construct a ceRNA network, we employed the approach summarized in Fig. S3A. Briefly, we used the sRNA toolbox, which includes Simple Seed Analysis, TargetSpy, miRanda, and PITA prediction software, to predict the targeting relationship between *N. bombycis* lncRNA and Nb-milRNA/bmo-miRNA. We then simultaneously selected relationship pairs that were predicted to be targeted in all four libraries. We then screened differentially expressed Nb-milRNAs and host bmo-miRNAs from the small RNA-seq datasets using thresholds of q < 0.01 and |log2(fold change)| > 1. Among the host miRNAs, only the significantly upregulated bmo-miRNAs were retained (Fig. S3b, c). Using the milRNA/miRNA set shared by both the target-prediction and differential expression results (Fig. S3d), we constructed an lncRNA-miRNA network (Fig. 2a). We used the sRNA toolbox to predict the mRNAs of *N. bombycis* targeted by these milRNAs/miRNAs and constructed a ceRNA regulatory network. Our analysis revealed that 18 *N. bombycis* lncRNAs were capable of affecting the regulation of 2514 mRNAs through competitive binding of five milRNAs and 20 miRNAs from the host.

ceRNA regulatory networks of NbLNCRNAs. Red markers are NbLNCRNA, and blue markers are BmmiRNAs/NbmilRNAs. (b) Construction of the *N. bombycis* pathogenicity-related ceRNA subnetwork based on NbLNCRNA. (c) Construction of the *N. bombycis* pathogenicity-related ceRNA subnetwork based on bmo-bantam-5p

We further screened the ceRNA regulatory network for genes involved in the pathogenicity of *N. bombycis*, including energy metabolism, invasion apparatus, and ricin lectin genes. This led to the construction of a sub-network of ceRNAs involved in NbLNCRNAs (Fig. 2b). Overall, 17 NbLNCRNAs were found to competitively bind to 19 miRNAs, thereby affecting downstream *N. bombycis* lectin-related genes, polar tube protein genes, spore wall protein genes, hexokinase genes, and ADP/ATP transporter protein genes. CytoHubba analysis identified bmo-bantam-5p as a key host miRNA node in this pathogenicity-related subnetwork. To further describe its topological position, we calculated its network parameters in the extracted subnetwork. In the deduplicated directed subnetwork, bmo-bantam-5p showed a degree of 14 and a normalized betweenness centrality of 0.00211, supporting its important topological position in the network. Therefore, we extracted the bmo-bantam-5p-centered ceRNA subnetwork for further analysis (Fig. 2c). bmo-bantam-5p was found to be one of the sub-branches of the *TCONS_00002914* (hereafter referred to as NbLNC2914) regulatory network, which may regulate several ricin lectin-related genes, trehalase genes, spore wall genes, polar tube protein genes, and other *N. bombycis* pathogenicity-related genes.

### 3.3 Identification and functional investigation of *NbLNC2914*

**Fig. 3:**
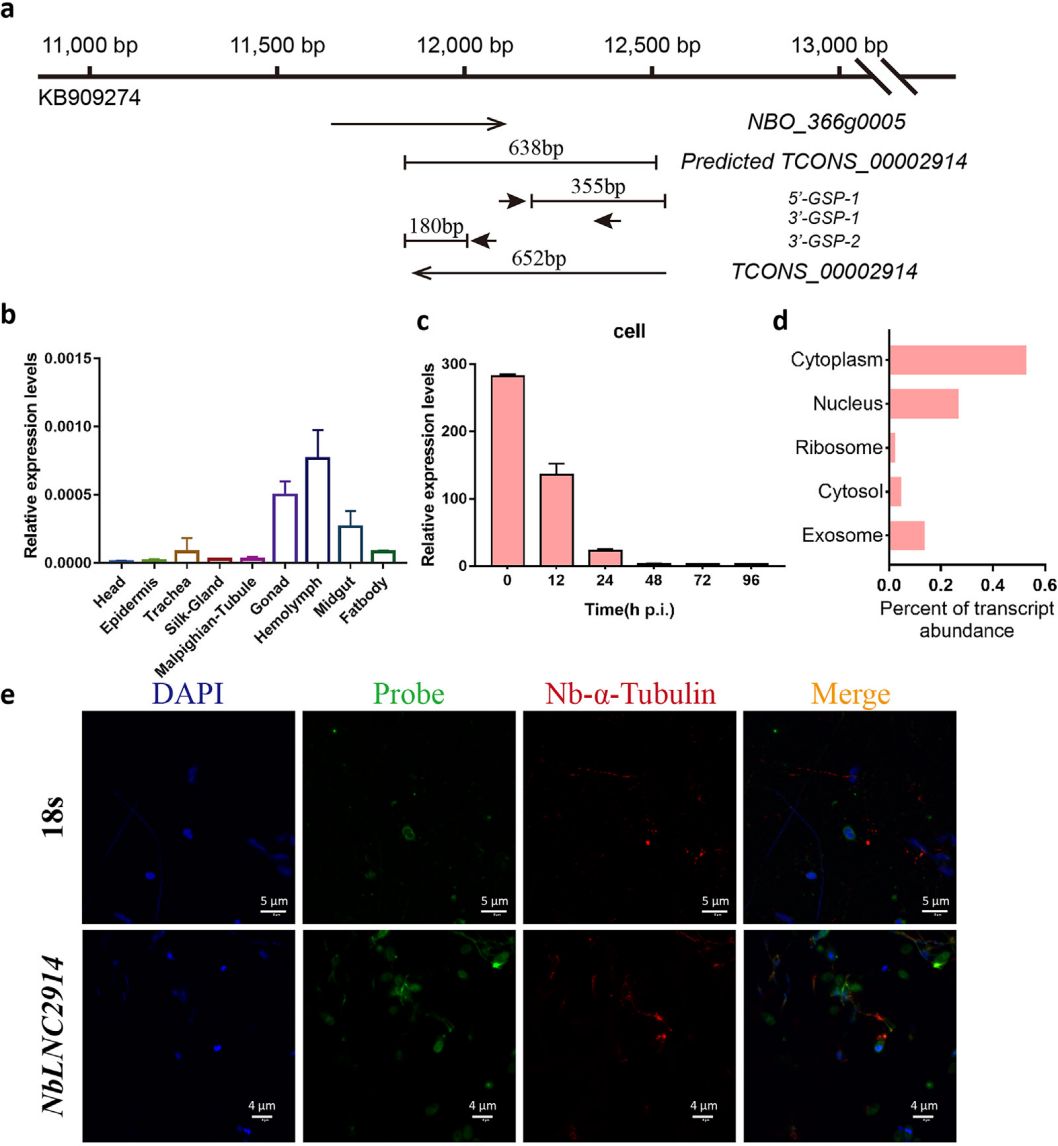
Identification and functional characteristics of *NbLNC2914*.

One of the lncRNAs identified in *N. bombycis, NbLNC2914*, consistently exhibited high expression levels after infection and had the highest weight in its mediated regulatory network among the ceRNA regulatory networks. This suggests that *NbLNC2914* plays a crucial role in the regulation of important biological processes in *N. bombycis. NbLNC2914*, which was found to be an overlapping transcript of *NBO_366g0005*, was localized to KB909274 in the *N. bombycis* scaffold (Fig. 3a). The full-length sequence of *NbLNC2914* was determined to be 652 bp through RACE amplification (Fig. S4a). The RNAfold WebServer was used to analyze the secondary structure of *NbLNC2914*, as well as the predicted minimum free energy and center-of-mass secondary structures (Fig. S4b). These structures exhibited typical characteristics of lncRNAs, such as a large number of loops, expanded parts, and helical regions.

(a) *NbLNC2914* and RACE primers at genomic positions. (b) Characteristics of *NbLNC2914* expression in various tissues after 3 days of infection in the silkworm. (c) Characteristics of *NbLNC2914* expression in infected cells. (d) Prediction of the subcellular localization of *NbLNC2914*. (e) Fluorescence in situ hybridization detection of *NbLNC2914* and 18 s. Amplification scale: 2 μm, other scale: 4 μm, DAPI staining indicates nuclei, green fluorescence indicates FISH probe, and red fluorescence indicates Nb-α-Tubulin protein.

To investigate the tissue-specific expression of *N. bombycis* lncRNA, we collected *B. mori* sample after *N. bombycis* infection at the day 1 5th instar larvae (5L1D), and sampled different tissues. qRT-PCR showed that *NbLNC2914* was specifically expressed in the midgut, gonads, and hemolymph tissues at the highest levels, followed by the tracheal and fat body tissues (Fig. 3b). We observed differences in the expression at different time points after infection with BmE-SWU1 cells. Specifically, *NbLNC2914* was highly expressed during spore germination and invasion, but showed decreasing expression over time and very low expression during the schizogony phase and maturation (Fig. 3c). To predict the subcellular localization of *NbLNC2914* in microsporidia, we used lncLocator, which indicated that it was more likely to be localized in the cytoplasm and nucleus (Fig. 3d). Our FISH experiments also demonstrated that *NbLNC2914* was mainly localized in the cytoplasm of *N. bombycis*, while it produced a weak fluorescent signal in the nucleus (Fig. 3e).

### 3.4 *NbLNC2914* acts as a miRNA sponge that inhibits the expression of bmo-miR-2808a-3p

**Fig. 4:**
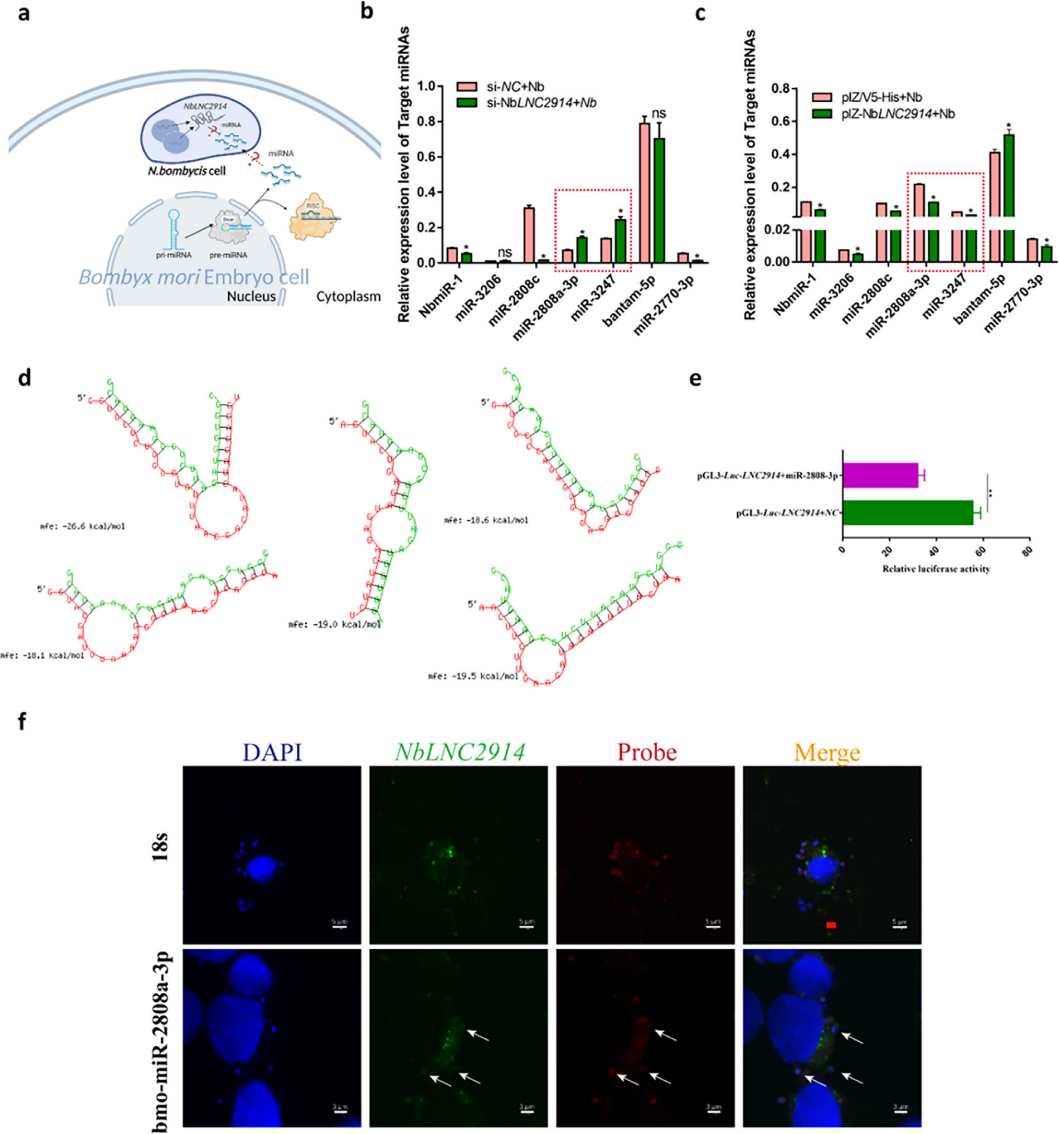
*NbLNC2914* acts as a miRNA sponge that inhibits the expression of bmo-miR-2808a-3p.

Cytoplasmic lncRNAs act as molecular sponges that bind to miRNAs and indirectly regulate their biological functions. *NbLNC2914* was expressed in *N. bombycis* cells and interacted with host miRNAs, indicating that it may regulate gene expression. It also shows how miRNAs are processed and transported in the host cell, with *NbLNC2914* influencing this process (Fig. 4a).

(a) The proposed model of *NbLNC2914* acting as a cytoplasmic molecular sponge for host miRNAs. (b) Expression levels of target miRNAs after *NbLNC2914* interference. (c) Expression levels of target miRNAs after *NbLNC2914* overexpression. (d) Prediction of binding between *NbLNC2914* and bmo-miR-2808a-3p based on RNAhybrid and further supported by miRanda analysis. (e) A dual-luciferase reporter system was used to evaluate the impact of bmo-miR-2808a-3p on the regulation of *NbLNC2914* recombinant luciferase activity. (f) Fluorescence in situ hybridization detection of the co-location of *NbLNC2914* and bmo-miR-2808a-3p Scale: 4 μm; DAPI staining indicates nuclei, green fluorescence indicates *NbLNC2914* probe, and red fluorescence indicates FISH probe (*p 〈 0.05; **p < 0.01; ns *p* 〉 0.05).

*NbLNC2914* was predicted to target NbmilRNA-1 and six *B. mori* miRNAs (miR-3206, miR-2808c, miR-2808a-3p, miR-3247, miR-bantam-5p, and miR-2770–3p). To verify the transcriptional regulation of NbmilRNA/BmmiRNA by *NbLNC2914*, we measured the expression of each miRNA by qRT-PCR using the pIZ-NbLNC2914 vector and the siRNA of *NbLNC2914* after up-regulation and repression of *NbLNC2914* expression, respectively, in the presence of *N. bombycis* infection with BmE-SWU1. The results showed that *NbLNC2914* expression significantly and negatively correlated with the expression of bmo-miR-2808a-3p and miR-3247 (Fig. 4b and c). Further analysis using RNAHybrid revealed that *NbLNC2914* has multiple binding sites for bmo-miR-2808a-3p (Fig. 4d). In addition, miRanda analysis further supported the interaction between NbLNC2914 and bmo-miR-2808a-3p, identifying a candidate binding site with a score of 157.0 and a minimum free energy of –20.94 kcal/mol under stringent settings (Fig. S5a). The dual-luciferase assay showed that miR-2808a-3p decreases the value of FLuc/RLuc in the wild-type group, indicating a negative regulatory effect of bmo-miR-2808a-3p on *NbLNC2914* (Fig. 4e). Moreover, the colocalization of *NbLNC2914* and bmo-miR-2808a-3p was detected using a FISH assay. bmo-miR-2808a-3p entered the *N. bombycis* sporoplasm after infection and colocalized with *NbLNC2914* (Fig. 4f). These results suggest that *NbLNC2914* binds to bmo-miR-2808a-3p and regulates its expression.

### 3.5 Expression characteristics and localization of bmo-miR-2808a-3p

**Fig. 5:**
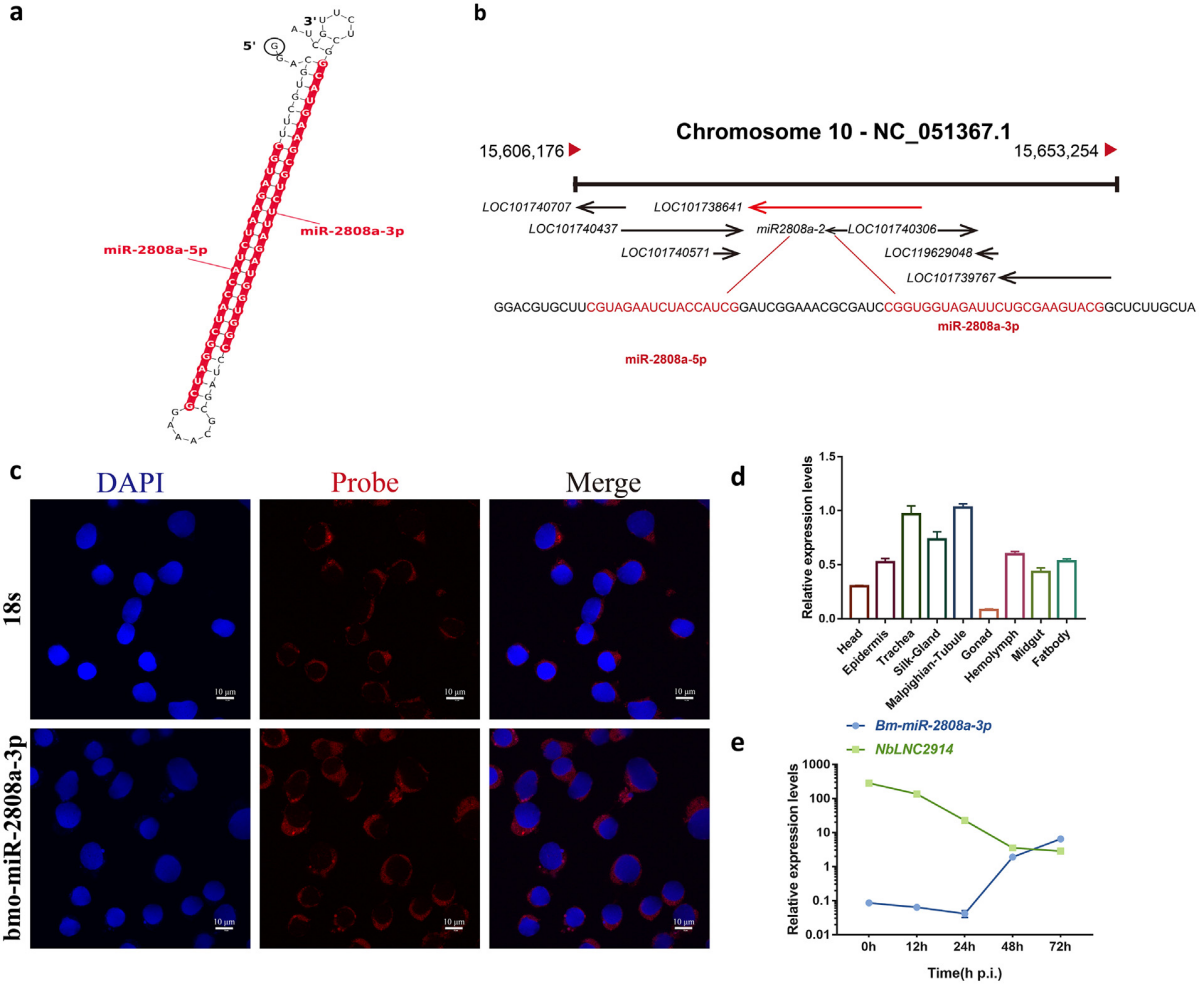
Expression characteristics and localization of bmo-miR-2808a-3p.

bmo-miR-2808a-3p is located on chromosome 10 of *B. mori*, transcribed from the intronic region of the *B. mori* protein-coding gene *melted* (NCBI accession number: LOC101738641) as the precursor miR-2808a-2, and subsequently processed from the 3′ stem-loop arm (Fig. 5a). Subcellular localization analysis demonstrated that bmo-miR-2808a-3p is predominantly expressed in the cytoplasm of BmE-SWU1 cells (Fig. 5c). To investigate the tissue-specific expression pattern of bmo-miR-2808a-3p after *N. bombycis* infection, various tissues of infected silkworms were sampled at 3 days post-infection. RT-PCR (qRT-PCR) analysis revealed that bmo-miR-2808a-3p exhibited the lowest expression levels in the gonads and the highest levels in the tracheal plexus and Malpighian tubules (Fig. 5d). Finally, total RNA was extracted from BmE-SWU1 cells at various time points post-infection to examine the temporal expression dynamics of bmo-miR-2808a-3p at different stages of *N. bombycis* infection. Expression levels were determined using poly(A)-tailed RT-qPCR. The results showed that bmo-miR-2808a-3p expression was very low in uninfected and early-stage infected cells, but increased significantly at 48 and 72 h. Interestingly, the expression pattern of bmo-miR-2808a-3p exhibited an inverse trend compared to that of *NbLNC2914* (Fig. 5e).

(a) Predicted secondary structure of bmo-miR-2808a. (b) Location of bmo-miR-2808a-3p in the silkworm genome. (c) Subcellular localization of bmo-miR-2808a-3p Scale: 10 μm; DAPI staining indicates nuclei, and red fluorescence indicates FISH probe. (d) Expression of bmo-miR-2808a-3p in silkworm tissues after *N. bombycis* infection. (e) Temporal expression of bmo-miR-2808a-3p and *NbLNC2914*.

### 3.6 bmo-miR-2808a-3p can regulate genes of *N. bombycis* across kingdoms

**Fig. 6:**
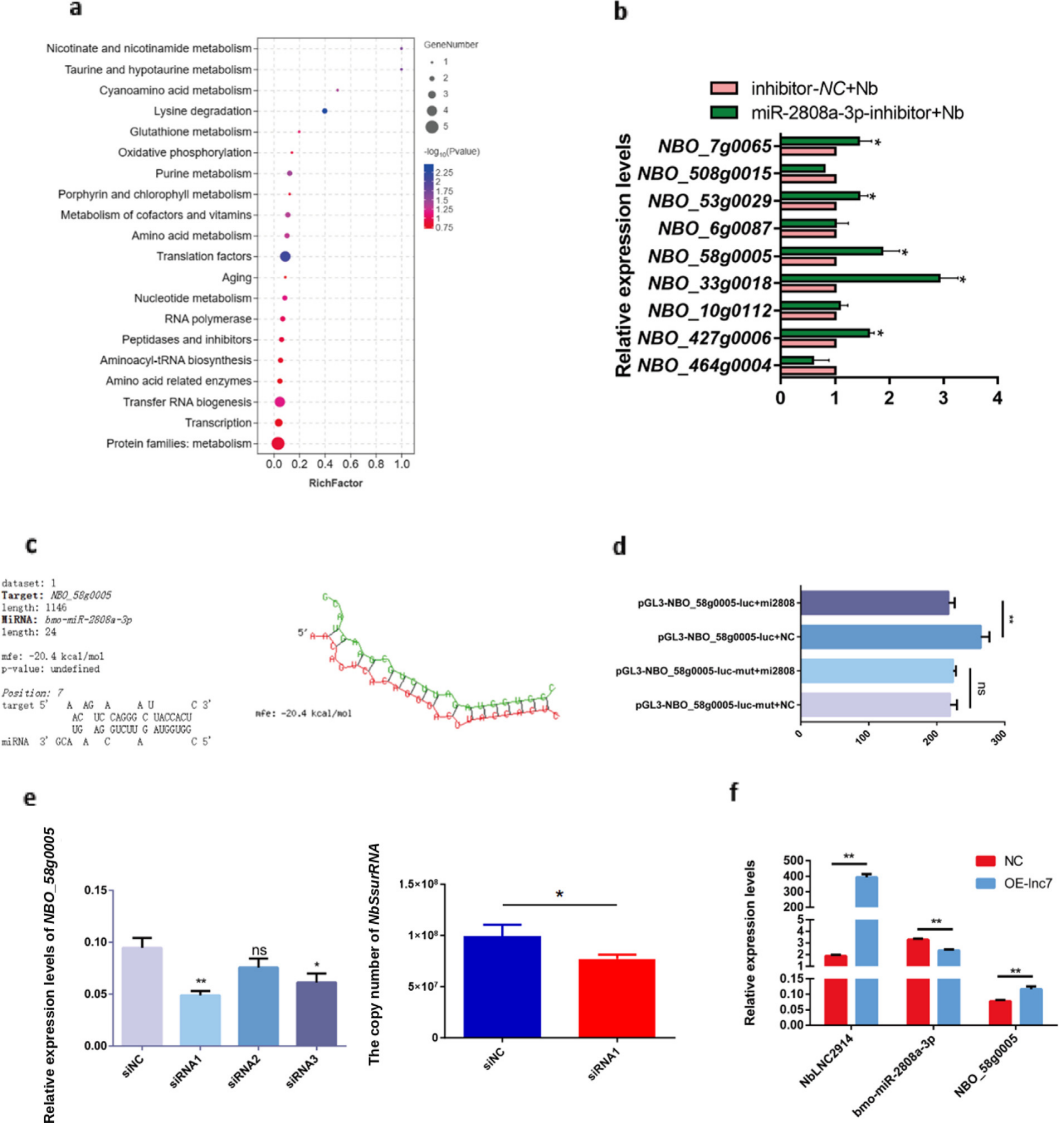
bmo-miR-2808a-3p can regulate *N. bombycis* genes across kingdoms.

To explore the regulatory effect of bmo-miR-2808a-3p on its downstream target genes, we predicted 65 potential target genes that were enriched in several metabolic pathways, including lysine degradation and genetic information processing pathways such as translation factors (Fig. 6a). KEGG enrichment analysis further revealed these enriched pathways. After inhibiting bmo-miR-2808a-3p in Bme cells, we observed significant upregulation of several target genes, indicating that inhibition relieved the repression of these genes (Fig. 6b). Among these, we focused on *NBO_58g0005*, which showed strong interactions with bmo-miR-2808a-3p The binding sites between bmo-miR-2808a-3p and *NBO_58g0005* were predicted through RNAHybrid analysis (Fig. 6c). We further evaluated this interaction using miRanda software. Although no site above the threshold was identified under stringent settings, a candidate binding site was detected under a moderately relaxed parameter setting, supporting a potential interaction between bmo-miR-2808a-3p and *NBO_58g0005* (Fig. S5b). Luciferase reporter assays were performed to confirm the interaction. The results showed that bmo-miR-2808a-3p significantly suppressed the expression of luciferase linked to the 3′ UTR of *NBO_58g0005* (Fig. 6d). In addition, we validated the functional relevance of *NBO_58g0005* using siRNA-mediated knockdown (Fig. 6e), which revealed that silencing *NBO_58g0005* inhibited *N. bombycis* proliferation. Moreover, overexpression of *NbLNC2914* resulted in decreased bmo-miR-2808a-3p levels and increased *NBO_58g0005* expression, further confirming the regulatory axis involving *NbLNC2914*, bmo-miR-2808a-3p, and *NBO_58g0005* (Fig. 6f).

(a) KEGG enrichment analysis of target genes of bmo-miR-2808a-3p (b) Expression of target genes after treatment with a bmo-miR-2808a-3p inhibitor. (c) Predicted binding between bmo-miR-2808a-3p and *NBO_58g0005* based on RNAhybrid analysis and further evaluated by miRanda. (d) Dual-luciferase reporter assay validating the regulation of *NBO_58g0005* recombinant luciferase activity by bmo-miR-2808a-3p (e) Verification the effect on the proliferation of N*. bombycis* after interference with *NBO_58g0005* gene. (f) Analysis of the regulatory relationship among *NbLNC2914*, bmo-miR-2808a-3p, and *NBO_58g0005*.

## 4 Discussion

Analysis of the genomic differences between *Encephalitozoon* and *Nosema* species revealed that the noncoding regions of their genomes are highly conserved and evolve more slowly than the protein-coding regions, indicating that the intergenic regions retained under selective pressure may serve essential core functions [34]. Early studies using 5′/3′ RACE-PCR identified and compared noncoding RNAs in *Encephalitozoon* and *Nosema*, revealing that 15 out of 18 snRNAs and snoRNAs from Encephalitozoon are also present in *Nosema* [35]. High-throughput sequencing and bioinformatics analyses have enabled preliminary exploration of noncoding RNA features and functions in microsporidia, but the targeting relationships and functional validation still require experimental support. In this study, we aimed to elucidate the roles and regulatory mechanisms of ncRNAs, specifically lncRNAs and milRNAs, during *N. bombycis* infection in *B. mori*. Using high-throughput sequencing of key infection stages (spore germination, schizogony, and spore maturation), we systematically identified and characterized 30 novel lncRNAs and 12 new milRNAs specific to *N. bombycis*. Moreover, we confirmed that *NbLNC2914* acts as a molecular sponge involved in modulating the host miRNA bmo-miR-2808a-3p and influencing target mRNA *NBO_58g0005* expression. These results significantly enhance our understanding of the molecular intricacies underpinning the life cycle of *N. bombycis* and host-pathogen interactions.

Previous studies have explored the roles and regulatory networks of lncRNAs, miRNAs, and mRNAs in interactions between *B. mori* and *N. bombycis.* Pu et al. [36] identified 1440 lncRNAs in the *B. mori* midgut following *N. bombycis* infection, highlighting 42 significantly differentially expressed lncRNAs (DElncRNAs). These DElncRNAs were shown to modulate host immune responses and metabolic pathways primarily via interactions with miRNAs. Notably, the lncRNA MSTRG857.1 was significantly upregulated during infection, possibly facilitating pathogen proliferation by suppressing host cell apoptosis [36]. This research provides a valuable molecular basis for understanding host-pathogen interactions and opens avenues for targeted therapeutic strategies against microsporidiosis. Chen et al. [37] investigated the regulatory interactions between lncRNAs, miRNAs, and mRNAs during silk gland apoptosis in *B. mori*. Their study characterized the extensive involvement of lncRNAs and miRNAs in the modulation of apoptosis at distinct developmental stages. These findings highlight miRNAs as more pivotal regulators than lncRNAs in the apoptosis process, establishing foundational insights into the regulatory mechanisms underlying developmental apoptosis in *Lepidoptera*. Shen et al. performed comprehensive pan-transcriptomic analyses involving both *N. bombycis* and *B. mori* during congenital infection and identified extensive networks of 403 lncRNAs, 62 circRNAs, and 284 miRNAs within the pathogen [21]. To accomplish this, they constructed elaborate ceRNA networks highlighting critical pathogen genes, such as *PTP3, ricin-B-lectin, SWP4*, and *HSP90*, implicating these networks in crucial infection processes. Comparatively, our study focused on the detailed mechanisms underlying pathogen–host interactions during specific stages of *N. bombycis* infection (spore germination, schizogony, and maturation phases). Unlike previous studies, our research explicitly identified and validated *NbLNC2914* as a ceRNA for bmo-miR-2808a-3p, which subsequently modulated the expression of *NBO_58g0005*. From an environmental perspective, host–pathogen cross-kingdom RNA regulation may represent a mechanism through which microsporidia adapt to fluctuating external conditions. Environmental stressors commonly encountered in sericultural systems, such as nutritional imbalances and microbial pressure, alter host small RNA expression profiles. Such changes can indirectly enhance ncRNA-mediated regulatory interactions, thereby facilitating microsporidian proliferation.

While the *Nosema apis* genome is 7.86 Mb, the *N. bombycis* genome is 15.7 Mb and includes longer intergenic regions [[38], [39], [40]], indicating that the noncoding RNAs identified in this study represent only a fraction of the noncoding RNAs in *N. bombycis*. A functional spectrum of lincRNAs constructed in *Schizosaccharomyces pombe* revealed that most lincRNAs play intracellular regulatory roles in response to specific environmental or physiological conditions [41]. In unicellular fungi, lncRNAs respond to environmental changes by regulating gene expression and are also involved in structural formation and pathogenicity [42]. Although studies on *Nosema apis* have provided insights into the involvement of lncRNAs in host infection, research on lncRNAs in microsporidia is limited. LncRNAs can act as molecular sponges to competitively bind miRNAs, forming ceRNA networks that regulate miRNA-targeted mRNA expression [43]. Studies on fungal or parasitic lncRNAs have predominantly relied on transcriptomic predictions, with few experimental validations [44]. Fungal lncRNAs typically respond to environmental stress, regulate gene expression, and participate in pathogenic processes [45]. However, research on microsporidia remains particularly scarce. Our study identified *NbLNC2914* as a crucial lncRNA that acts as a sponge for bmo-miR-2808a-3p, thereby modulating the expression of the *NBO_58g0005* gene. This is the first experimentally validated instance of a microsporidian lncRNA-miRNA-mRNA regulatory axis, underscoring the complexity and functional significance of ncRNA-mediated regulatory mechanisms in microsporidian biology. This discovery expands the current understanding beyond previously recognized miRNA-mRNA interactions, highlighting the essential regulatory roles of lncRNAs in microsporidian pathogenicity and proliferation. Although our results support the interaction between NbLNC2914 and host miRNAs in the host cytoplasmic environment, the mechanism by which parasite-derived lncRNAs reach this compartment remains unclear. Currently, there is a lack of direct experimental evidence for the delivery of microsporidian lncRNAs into host cells. Based on the established invasion process of microsporidia, a plausible explanation for this is that NbLNC2914 is co-transported with sporoplasm components into the host cytoplasm through the polar tube during spore germination. Moreover, sporoplasm-associated proteins have been shown to interact directly with host organelles during early infection, supporting the idea that parasite molecules delivered during invasion can rapidly engage host intracellular components. However, these possibilities remain hypothetical and require direct experimental validation in future studies [46]. In order to determine whether this pathway functions consistently under physiological infection conditions, future studies should further verify the biological significance of the NbLNC2914-bmo-miR-2808a-3p-NBO_58g0005 regulatory axis in vivo using silkworm infection models. Given the promotive role of NbLNC2914 in *N. bombycis* proliferation, it is of interest to explore whether targeted inhibition of NbLNC2914 could provide a potential avenue for controlling microsporidian infection. In addition to regulatory network analysis, we conducted preliminary experiments to explore potential protein partners that interact with NbLNC2914 (Fig. S6). Although these initial results suggest that NbLNC2914 may associate with specific proteins in *N. bombycis*, we did not further characterize the identities or functional roles of these interacting proteins within this study. Future work will focus on systematically identifying and validating NbLNC2914-binding proteins, as well as elucidating the molecular mechanisms by which lncRNA-protein complexes contribute to parasite development and host-pathogen interactions. These investigations will further expand our understanding of the diverse roles of lncRNAs in microsporidian biology and may reveal new targets for interventions.

## 5 Conclusions

In this study, we identified 30 novel lncRNAs and 12 new milRNAs in *N. bombycis*, constructed an NbLNC-based ceRNA regulatory network, and provided valuable resources for investigating ncRNA-mediated regulatory mechanisms in microsporidian biology. Functional analysis of NbLNC2914 confirmed its role as a molecular sponge involved in modulating host miRNAs and influencing target mRNA expression. These findings offer new perspectives on host-microsporidia interactions and establish a theoretical basis for further elucidation of the mechanisms of microsporidia infection and resistance development.

## Data availability statement

All discussed data are included in the manuscript or in the Supplementary Information. The RNA-seq data generated and analyzed in this study are available from the NCBI: https://www.ncbi.nlm.nih.gov/bioproject/PRJNA1273716.

## CRediT authorship contribution statement

**Pengcheng Zhang:** Writing – original draft, Visualization, Validation, Methodology, Data curation, Conceptualization. **Boyuan Deng:** Writing – original draft, Methodology, Data curation. **Wenxuan Fang:** Methodology. **Feifei Liu:** Visualization. **Peng Chen:** Supervision. **Xuhua Huang:** Methodology. **Minhui Pan:** Writing – review & editing, Supervision, Project administration, Funding acquisition. **Zhanqi Dong:** Writing – review & editing, Supervision, Project administration.

## Declaration of competing interest

The authors declare that they have no known competing financial interests or personal relationships that could have appeared to influence the work reported in this paper.

## Appendix Supplementary materials

Image, application 1

Image, application 2

Image, application 3

Image, application 4
