# Supplementary material for: Cross-kingdom noncoding RNA regulation facilitates Nosema bombycis proliferation
Source: Eng Microbiol. 2026 Jun 3;6(3):100278. doi: 10.1016/j.engmic.2026.100278 (PMC13276322; doi:10.1016/j.engmic.2026.100278)
Supplement: Supplementary file 1 [file mmc1.docx]

**Table S1 The table of lncRNA and sRNA data quality control**

The table of lncRNA data quality control

| Sample | Raw_reads | Clean_reads | Q20(%) | Q30(%) | GC(%) |
| --- | --- | --- | --- | --- | --- |
| NB_12h | 75,549,278 | 73,148,502 | 0.9858 | 0.9578 | 0.5096 |
| NB_48h | 74,563,848 | 72,674,082 | 0.9854 | 0.9567 | 0.5046 |
| NB_96h | 71,824,362 | 69,251,438 | 0.9851 | 0.9558 | 0.5054 |
| CT_12h | 76,468,538 | 73,810,530 | 0.9847 | 0.9552 | 0.5064 |
| CT_48h | 77,368,860 | 74,652,984 | 0.985 | 0.9551 | 0.5076 |
| CT_96h | 69,275,652 | 67,255,708 | 0.9844 | 0.954 | 0.5096 |

The table of sRNA data quality control

| Sample | Total reads | Clean_reads | Q20(%) | Q30(%) | GC(%) |
| --- | --- | --- | --- | --- | --- |
| CT_12h | 28,622,499 | 27,954,980 | 84.72% | 79.84% | 75.52% |
| CT_48h | 19,050,224 | 18,617,137 | 89.97% | 84.25% | 72.48% |
| CT_96h | 24,659,204 | 23,819,096 | 92.10% | 86.64% | 71.53% |
| NB_12h | 26,242,556 | 25,538,175 | 85.08% | 80.18% | 74.20% |
| NB_48h | 35,250,686 | 34,082,762 | 91.81% | 86.11% | 71.67% |
| NB_96h | 20,957,270 | 20,379,411 | 89.00% | 83.29% | 71.44% |
