# Supplementary material for: Cross-kingdom noncoding RNA regulation facilitates Nosema bombycis proliferation
Source: Eng Microbiol. 2026 Jun 3;6(3):100278. doi: 10.1016/j.engmic.2026.100278 (PMC13276322; doi:10.1016/j.engmic.2026.100278)
Supplement: Supplementary file 2 [file mmc2.docx]

**Table S2 Predicted lncRNAs in *Nosema bombycis***

| Name | Locus | Sequence |
| --- | --- | --- |
| TCONS_00000007 | – | CAACAATCCAACAAACCAATCAGAGCTACCGACAACTTTAGGGGCTCATCTTCAAACAACAACAAATCTAATAAAAACATTAAAACCAATTCAAATATAAACATTCTGGAGACGTATCTCCATGAGGGATACGTCAGTTTGTTAACAAAGAGCGTAACCACAAAGAGTGGTCTCCAGAGCAGAACAAGATTGATACAGAAATTTGATAAAAATAAAAATCCAAAAAAGATTAAATTCAAGAAACCGTTCGAGGGAATTGTAGACGATTGGTACGAAGAGGTATCAATAATAGAAGATATAAGTGGCATAGAAGTTTTTGAATGGTGCGAATTATTCGAAGATTTATCAATGACAAACAAGTGGAACAAGTCGGTGCAGATACAATTATTAAAGAGATTAGTCAAAGATCCCATAATAACGGATAGAGGACTTAAAGAGTCCTGGAAAGAAATTAGAAGACAGTTAATAACCGCAGTGTACCCAGAGG |
| TCONS_00000008 | – | AGTAATAGTATTCTTTCATAATTATAAAACATTTTGATAATTGTACTGCCATGGGATCAATAGGATGTCATAACGATGAAGAACATAAAAGAATATGATAAAACATAGTCTTTGAATTCTAATTCATTTTAGGATAACCCTTTGAACTTAAGCATATCAGTAAAAGGAGGAAAAGAAACTAACAAGGATTTCTTTAGTAGTGGCGAATGAACAAGAAAGAACTCATTATGTAATCTATATTCCAGTGGTTCCCAACCTTTTTAGGTTCGCGCCCCACTTTCGGGATTTTGAAATCAAAAATGCCCCCTTGCCC |
| TCONS_00000141 | 1 | AAGCAAATCATCCTAAGCTCTCTTTTGTTTATAAGTAGCATCTATACAAGTAATGTAAATGGTAAGAACCTTTCTGTATTCGGTGCCTTTTTCGGATCACCTAACGATAATGATACCACTCGTGAACACTGTTGTAGTCCCCGTAACAAATTTCATCTTACGAGCTTTAGTTCTGGATGTGATCTTCAAAAAGAAGTTGAGTGTCTTAGAGCTGAATGTAAAAAATCCACAGAATGTTGCAATGTCGTCTCTTTGTGTTCTGGACTTAGTGAATGCGAATACGTTGAAAAAACTTGCGATGCCATAAAACAATACTACCAAAAACAAGCAATAAACACGGACGTAAAGATTACATTAGTCTTAAAATGGATTTACAAATGTATGAACACTACTTCTAGGGCTAAGTTGGCCGTATTCACTGCTCATGCCATTTTCAATACTAGTGCATTTGCATGCCTTGAAGCCGAAGGCACCTGGAAATACAGATCAAGAGGTTTACTTGCTATCCAAGGAGAAAAGAACTATGGGCTTTTAACTTCTTACTCTAGAAACCGTGAAAATTTCAAAGAATGTCCTCATCGTTTGGCTGACTTAAATTGTGATGTCATCAGTGTTACTGTCGACTGGTGGTACAGAAATGTAGGAAAGTGTTGTAAAGATTTCTTCGGAAGTCTTGAGATTCTAAAGCCTTCCGAATGGACTGCTTTAAAGAAAAACTGTGCCGATAGGGAGTCTGCTAGAAGATTAGAAAACAGAAGAAGATTGTACGAAATATTGGTTGGATG |
| TCONS_00002914 | – | AGGTGCTCTGGTGTGTTTAACGACATACTACCACCTCACCTCCTGAGAAGAAAGAAGAAGTACTGAGATGAGACTATCTAATTGGCAGATGAAATAGGATTAAAGAAGATAATGAATGTTAAGTGTATGTAAGACAGGAAAAGAATGTACGATTAAAACGGGAAAACTAAAAGCCCAATGTGTGAGGAGACCCTAAGAGGCTCAGAGAGAGAGAGACGGTACGATTTAAAAGGGGAAAGGACAGCCAGAGAGAGCGCGCCCCAGAGGACTCCAGAGAGAGGAGACGGTACGATTTAAAGGGGATAACAGATGGCCCAGAGTGTGAGGCGCACCCCAAGGAACTCAGAGAGAGACACAGTACAATTTATGTAAAAACCACAACTAGATGGTTGATGGATTCCTTAGCTAATAGGACCATCAAAACTTTTCTTCCTATAAATTAGGGTTAGCAGCTATAACTAACGTCTTAACATCACTTAACAACCCCCCCTCCACCATGTTTTCATTATCTTTTTTCAATTCTTTTTCTTTTGTCATTTGTATATTTTTGGATTGGTCAGCACAAGTGGAGGAAGTTGGTCCCAAGAACTTCTTGAAGATAGAGTCTAGTAAAATAGAAGATTTGAGAAGAAAATAAAAGTAATTACCAACA |
| TCONS_00000152 | 1 | AGCAAATCATCCTAAGCTCTCTTTTGTTTATAAGTAGCATCTATACAAGTAATGTAAATGGTAAGAACCTTTCTGTATTCGGTGCCTTTTTCGGATCACCTAACGATAATGATACCACTCGTGAACACTGTTGTAGTCCCCGTAACAAATTTCATCTTACGAGCTTTAGTTCTGGATGTGATCTTCAAAAAGAAGTTGAGTGTCTTAGAGCTGAATGTAAAAAATCCACAGAATGTTGCAATGTCGTCTCTTTGTGTTCTGGACTTAGTGAATGCGAATACGTTGAAAAAACTTGCGATGCCATAAAACAATACTACCAAAAACAAGCAATAAACACGGACGTAAAGATTACATTAGTCTTAAAATGGATTTACAAATGTATGAACACTACTTCTAGGGCTAAGTTGGCCGTATTCACTGCTCATGCCATTTTCAATACTAGTGCATTTGCATGCCTTGAAGCCGAAGGCACCTGGAAATACAGATCAAGAGGTTTACTTGCTATCCAAGGAGAAAAGAACTATGGGCTTTTAACTTCTTACTCTAGAAACCGTGAAAATTTCAAAGAATGTCCTCATCGTTTGGCTGACTTAAATTGTGATGTCATCAGTGTTACTGTCGACTGGTGGTACAGAAATGTAGGAAAGTGTTGTAAAGATTTCTTCGGAAGTCTTGAGATTCTAAAGCCTTCCGAATGGACTGCTTTAAAGAAAAACTGTGCCGATAGGGAGTCTGCTAGAAGATTAGAAAACAGAAGAAGATTGTACGAAATATTGGTTGGATG |
| TCONS_00000968 | – | AATTTACCAGGTATAACATGGTGTAATATTTTATCATGATAGTGGTGCATGGCCGTTTCCAATGGATGCTGTGAAGTAATGATTAATTTCAACAAGATGTGAGACCCTCATTTAGACAGATGTAGTGATACATATGAAGGAGAGGATTAAAACAGGTCCGTTATGCCCTAAGATAATCTGGGTTGCACGCGCAATACAATAATATTTGATATTATAAGGGATAATATAATGTAAGATATATTTGAACACGGAATTGCTAGTAAATTTTATTTAATAAGTAGAATTGAATGAGTCCCTGTTCTTTGTACACAC |
| TCONS_00001630 | 2 | GGAGATTACCAGATGACAGTATTAAAGAATCATAGGTGATTTGTCATCTGGTAAGACCCGAAACATAGTGAACTATATATGTCAATGGTTGAAGATAAACGACCGTTTATTGGAAGACCTAAGTCATTCTGACGTGCAAATCGATGGTGAAAGATGTATATAGTGGCGAAAGACCAATCGAACTATGTGGTAGCTGGTTCACAGCGAAATGTCTCTTAGGACAGTTGAGTATAAATAAAGTAAATAGATGTAGAGTCAATACAATGTTTAATTATATTGTTTAACTTCTAATTCTATAGGTTTGTATGTTTTATAAATACTTACTGATATTTACGATACTCAAGTGGGCAAGAATAAGATCTATCAGTTAGTTGTTAAGGTAATGGCTTAACAAGACTATGACGGATAACGGTATTACTTTGTAATATTCCGGAGAAGGAGCCTGAGAGATTGCTACTAAGTCTAAGGATTGCAGCAGGGGCGAAACTTGACCTATGATATTATATTGAGGCAGTTATGAGTAGTATTTTATAATTATTGTAGTATTGTAAGTACATATTACAAGATAAATCGGAGGGCAAATCGAGTGCCAGCAGCCGCGGTAATACTTGTTCCGATAGTGTGTATGATGATTGATGCAGTTAAAAAGTCTGTAGTTTATTTATAATAAGCATTGTAAGGTATACTGTATGGTTAGGAGAGAGATGAAATGTGATAACCCTAACTGGATGAACAGAAGCGAAAGCTGTATACTTAAATGTATTATTAGAACAAGGACGTAAGCTAGAGGATCGAAGATGATTAGATACCATTGTAGTTCTAGCAGTAAACTATGTTGAATCATAGATATATTTTGATATATATTTATGTAGAGAAATTAAGATTATATTGATTCTGGGG |
| TCONS_00002520 | – | TTCAAATTATCTATATATTAAATACGTGAAGCAAAAAACTTTGTATTCCTTTTTACGAAAATTGCGCGGACGGAGGAGTATGAAATTTTCCACACTTATAGAAGATATAGAGAAGAAGTGCACAATGCTAATATTTATTTTAAATAATGCGTAAAAGATACATTAAATTAATAAAGAAAACATTATACACACTACCATGTATTTGATACACACACGCATGTATACTATTTGT |
| TCONS_00002522 | – | TATCACATGATTTTTTTTTTGGAACGAAGTTCCCTAACCGGGAAAAAACGTCCGTAACGTAAGATTTTTATTAGTAATGCACACAGTGTACGACTTAACTTTGTAATAACGTACAAAAAATAACATATTTTTTTATCATTCATTGACCACGATCTCAGAGCGTTCGTTTGCGCAATACACTAACTCTTATGCAAACAACCGTGAATGAAGTGTACCACAATAAGTTCGCACGTTACCGAATGACCGTGGGTTGTTGCGAAACCTCCAGTTTTATTTTCTTTATACCTCGAATAGAACTTAAAATTAATATTTTTATAATAAGGAACTTCGTTCCTATCCGGTGTCCCACGACACCACACATCTTTTTTATGGTTATTTTC |
| TCONS_00002524 | 3 | ACTTCTATAATCGCAGATTTCGCCAAGACCACGTTTTACACAAATATTAATAAAGACAAACAATATTTAATCTATTCTCAATTTGACCACAGATTTTAAGCAGTAAACTTGATGACTAAAAACTTGACAATATACAAATAGTATACATGCGTGTGTGTATCAAATACATGGTAGTGTGTATAATGTTTTCTTTATTAATTTAATGTATCTTTTACGCATTATTTAAAATAAATATTAGCATTGTGCACTTCTTCTTAGTTCTCTATAAGTGTGGAAAATTTCATACTCCTCCGTCCGCGCAATTTTCGTAAAAAGGAATACAAAGTTTTTTGCTTCACGTATTTAATATATAGATAATTTGAAA |
| TCONS_00002525 | 3 | AATTAGTAAGACTAGTGGCCCCCGGTGGTCGAAATTCGACTATAATGAGGTGAAATTAGGTATAAGTTTGTACACTATCATGATTCTATTGTCAAAGACTTCTATAATCGCAGATTTCGCCAAGACCACGTTTTACACAAATATTAATAAAGACAAACAATATTTAATCTATTCTCAATTTGACCACAGATTTTAAGCAGTAAACTTGATGACTAAAAACTTGACAATATACAAATAGTATACATGCGTGTGTGTATCAAATACATGGTAGTGTGTATAATGTTTTCTTTATTAATTTAATGTATCTTTTACGCATTATTTAAAATAAATATTAGCATTGTGCACTTCTTCTCTATATCTTCTATAAGTGTGGAAAATTTCATACTCCTCCGTCCGCGCAATTTTCGTAAAAAGGAATACAAAGTTTTTTGCTTCACGTATTTAATATATAGATAATTTGAAA |
| TCONS_00002526 | 3 | AATTAGTAAGACTAGTGGCCCCCGGTGGTCGAAATTCGACTATAATGAGGTGAAATTAGGTATAAGTTTGTACACTATCATGATTCTATTGTCAAAGACTTCTATAATCGCAGATTTCGCCAAGACCACGTTTTACACAAATATTAATAAAGACAAACAATATTTAATCTATTCTCAATTTGACCACAGATTTTAAGCAGTAAACTTGATGACTAAAAACTTGACAATATACAAATAGTATACATGCGTGTGTGTATCAAATACATGGTAGTGTGTATAATGTTTTCTTTATTAATTTAATGTATCTTTTACGCATTATTTAAAATAAATATTAGCATTGTGCACTTCTTCTCTATATTAGTTCTCTATAAGTGTGGAAAATTTCATACTCCTCCGTCCGCGCAATTTTCGTAAAAAGGAATACAAAGTTTTTTGCTTCACGTATTTAATATATAGATAATTTGAAA |
| TCONS_00002816 | 4 | ATAATGTGTCTTCTTTTTATCCACCTTATCCCTCTAGGTGAGGTCGGCACAGCAATTTTTTCTCTTCCATTCTCTTCTATCAGCCGTCATCTCAACACTCACTCCTCTCTCTCTCATATCGTCATTCACACACTCCATCCATGTCTTCTTCGGTCGATCTCTTCCCCCTCTACCTTGCACTACCATTTCCATACATCTCCTAGTCACATGCATCTTTTTTTTTTTGTTTTTTATTGCTTAGATGGGTAGACGAGCTCACAGCCCACCTGGTGTTAAGTGGTTACTGGAGCCCATAGACATCTACTACGTACATGCGCCACCCATCTCGAGGTATAAGTTCTAAGGTCTCAGTATAGTTACAACGGTTGCCCCACCCTTCAAACCGAAACGCATTACTGCTTCACGGCAGAAACAGGCGGGTTGCTGGTACCCACCCGTGCGGACTCACAAGAGGATTTACCACCAGTAATTAAGAAAA |
| TCONS_00002817 | 4 | TCTTTCAAGCCGTATGAAGTCGTCGTGACCTAAAGGATAAGACGTCCGGTGCATTCGTATGTAGCGATGCACCGGTGTTCGAATCCCGCAGGCGGGTACCAATTTTTCTAATGAAATACGTACTTAACAATTGTTCACGATTGACTTCCACGGTGAAGGAATAACATCGTGGTGTATGGTGGTAGGACCTCTTGTGAGTCCGCGCGGGTAGGTACCACCGCCCCGCCTATTACTGCCGTGAAGCAGTAATGCGTTTCGGTTTGAAGGGTAGGGCAGCCGATGTAACTATATTGTAGATGTCTATGGGCTCCAGTAACCACTTAACACCAGGTGGGCTGTGAGCTCGTACACCCACCTAAGCAATAAAAAAAACAAGCGCAACGTGTGAATGTGTTGAACGCGAGCTACATGGTAGGCGGAGTGGGGGTGTTAGGATTTATTTTCGTTACGGAATTCCTAGACTCGGTCGCCACACTCAAAGCACGCGATAAAAGCTATGCAATAGCTTAAAAAGTA |
| TCONS_00002818 | 4 | TCTTTCAAGCCGTATGAAGTCGTCGTGACCTAAAGGATAAGACGTCCGGTGCATTCGTATGTAGCGATGCACCGGTGTTCGAATCCCGCAGGCGGGTACCAATTTTTCTAATGAAATACGTACTTAACAATTGTTCACGATTGACTTCCACGGTGAAGGAATAACATCGTGGTGTATGGTGGTAGGACCTCTTGTGAGTCCGCGCGGGTAGGTACCACCGCCCCGCCTATTACTGCCGTGAAGCAGTAATGCGTTTCGGTTTGAAGGGTAGGGCAGCCGATGTAATTTTTTTTTGTTTTTTATTGCTTAGATGGGTAGACGAGCTCACAGCCCACCTGGTGTTAAGTGGTTACTGGAGCCCATAGACATCTACTACGTACATGCGCCACCCATCTCGAGGTATAAGTTCTAAGGTCTCAGTATAGTTACAACGGTTGCCCCACCCTTCAAACCGAAACGCATTACTGCTTCACGGCAGAAACAGGCGGGTTGCTGGTACCCACCCGTGCGGACTCACAAGAGGATTTACCACCAGTAATTAAGAAAA |
| TCONS_00002819 | 4 | TCTTTCAAGCCGTATGAAGTCGTCGTGACCTAAAGGATAAGACGTCCGGTGCATTCGTATGTAGCGATGCACCGGTGTTCGAATCCCGCAGGCGGGTACCAATTTTTCTAATGAAATACGTACTTAACAATTGTTCACGATTGACTTCCACGGTGAAGGAATAACATCTATACTAATATATAAATCTACAGTGGTTTTTACGGATGTTCCGTTATAACTACTGAACCATGCATCCGATTGACTTGAAACTTTGTATCCATGTAGAAAATACATGTACTTAATGGATAGGCTAATATTTATATGAGTGTTGGACTCCCTACACCAGTTGCGGGGGCGTTAATGATGAGAATCTTTGTGGGGGTGAGAAATAATAATGTTAATTTTAAATGCCCAGCGAAGCGGACGGGTACAGCTAGTATC |
| TCONS_00002820 | 4 | TCTTTCAAGCCGTATGAAGTCGTCGTGACCTAAAGGATAAGACGTCCGGTGCATTCGTATGTAGCGATGCACCGGTGTTCGAATCCCGCAGGCGGGTACCAATTTTTCTAATGAAATACGTACTTAACAATTGTTCACGATTGACTTCCACGGTGAAGGAATAACATCGTGGTGTATGGTGGTAGGACCTCTTGTGAGTCCGCGCGGGTAGGTACCACCGCCCCGCCTATTACTGCCGTGAAGCAGTAATGCGTTTCGGTTTGAAGGGTAGGGCAGCCGATGTAACTATATAAATTATATCTCAAGGTGTGTGGCGCATTTACATTGTAGATGTCTATGGGCTCCAGTAACCACTTAACACCAGGTGGGCTGTGAGCTCGTACACCCACCTAAGCAATAAAAAAAATTAAAAAAAATGGCAAAATACATGTACTTAATGGATAGGCTAATATTTATATGAGTGTTGGACTCCCTACACCAGTTGCGGGGGCGTTAATGATGAGAATCTTTGTGGGGGTGAGAAATAATAATGTTAATTTTAAATGCCCAGCGAAGCGGACGGGTACAGCTAGTATC |
| TCONS_00002821 | – | AATCTTACCCATAAAAAAGACGTGTGGCACTCGGGGACTGCCGCGATAAAGCTATTGCAAAACATTTTTTATCAACTTATGCAATTACAATTAGACAATAATAATTTAATATTAAAACAATAATAAAAATAAGACCACGCTATATTTATAAACATTAACAAAAGCAAAACATTAACTGTCCCGTTCACACTCATAAGCTAGACCGCGCGAGAGAGAGATGGGCAGACTTTTCATGATGCGCATGCAGTGCGACGTCACGCCGCGCGCTTATTCACAAACACTACACAAGCGCAACGTGTGAATGTGTTGAACGCGAGCTACATGGTAGGCGGAGTGGGGGTGTTAGGATTTATTTTCGTTACGGAATTCCTAGACTCGGTCGCCACACTCAAAGCACGCGATAAAAGCTATGCAATAGCTTAAAAAGTTACTGAACCATGCATCCGATTGACTTGAAACTTTGTATCCATGTAGAAAATACATGTACTTAATGGATAGGCTAATATTTATATGAGTGTTGGACTCCCTACACCAGTTGCGGGGAAATAATAATGTTAATTTTAAATGCCCAGCGAAGCGGACGGGTACAGCTAGTATC |
| TCONS_00002825 | – | TTAAGCTATTGCATAGCTTTTATCGCGTGCTTTGAGTGTGGCGACCGAGTCTAGGAATTCCGTAACGAAAATAAATCCTAACACCCCCACTCCGCCTACCATGTAGCTCGCGTTCAACACATTCACACGTTGCGCTTGTGTAGTGCGCATCATGAAAAGTCTGCCCATCTCTCTCTCGCGCGGTCTAGCTTATGAGTGTGAACGGGACAGTTAATGTTTTGCTTTTGTTAATGTTTATAAATATAGCGTGGTCTTATTTTTATTATTGTTTTAATATTAAATTATTATTGTCTAATTGTAATTGCATAAGTTGATAAAAAATGTTTTGCAATAGCTTTATCGCGGCAGTCCCCGAGTGCCACACGTCTTTTTTATGGGTAA |
| TCONS_00002970 | 2 | GTTGATTCTGCCTGACGTAGACGCTATACTCTAAGATTAACCCATGCATGTTTATTGAATATAAAGAAAAGACGAACAGCTCAGTAACTCTTATTTGATTTGATGTATTAGGATTCTAACTATGTTAAATTATAGGTAACAATAATACAATAAGAATAAGATCTATCAGTTAGTTGTTAAGGTAATGGCTTAACAAGACTATGACGGATAACGGTATTACTTTGTAATATTCCGGAGAAGGAGCCTGAGAGATTGCTACTAAGTCTAAGGATTGCAGCAGGGGCGAAACTTGACCTATGATATTATATTGAGGCAGTTATGAGTAGTATTTTATAATTATTGTAGTATTGTAAGTACATATTACAAGATAAATCGGAGGGCAAATCGAGTGCCAGCAGCCGCGGTAATACTTGTTCCGATAGTGTGTATGATGATTGATGCAGTTAAAAAGTCTGTAGTTTATTTTATAATAAGCATTGTAAGGTATACTGTATGGTTAGGAGAGAGATGAAATGTGATAACCCTAACTGGATGAACAGAAGCGAAAGCTGTATACTTAAATGTATTATTAGAACAAGGACGTAAGCTAGAGGATCGAAGATGATTAGATACCATTGTAGTTCTAGCAGTAAAC |
| TCONS_00003738 | 2 | CCAAGGAGATTACCAGATGACAGTATTAAAGAATCATAGGTGATTTGTCATCTGGTAAGACCCGAAACATAGTGAACTATATATGTCAATGGTTGAAGATAAACGACCGTTTATTGGAAGACCTAAGTCATTCTGACGTGCAAATCGATGGTGAAAGATGTATATAGTGGCGAAAGACCAATCGAACTATGTGGTAGCTGGTTCACAGCGAAATGTCTCTTAGGACAGTTGAGTATAAATAAAGTAAATAGATGTAGAGTCAATACAATGTTTAATTATATTGTTTAACTTCTAATTCTATAGGTTTGTATGTTTTATAAATACTTACTGATATTTACGATACTCAAGTGGGCAAGAATAAGATCTATCAGTTAGTTGTTAAGGTAATGGCTTAACAAGACTATGACGGATAACGGTATTACTTTGTAATATTCCGGAGAAGGAGCCTGAGAGATTGCTACTAAGTCTAAGGATTGCAGCAGGGGCGAAACTTGACCTATGATATTATATTGAGGCAGTTATGAGTAGTATTTTATAATTATTGTAGTATTGTAAGTACATATTACAAGATAAATCGGAGGGCAATCGAGTGCCAGCAGCCGCGGTAATACTTGTTCCGATAGTGTGTATGATGATTGATGCAGTTAAAAAGTCTGTAGTTTATTTTATAATAAGCATTGTAAGGTATACTGTATGGTTAGGAGAGAGATGAAATGTGATAACCCTAACTGGATGCAACAGAAGCGAAAGCTGTATACTTAAATGTATTATTAGAACAAGGACGTAAGCTAGAGGATCGAAGATGATTAGATACCATTGTAGTTCTAGCAGTAAACTATGTTGAATCATAGATATATTTTGATATATATTTATGTAGAGAAATTAAGATTATATTGACTCTGGGGATAGTATGATCGCAAGATTGAAAATTAAAGAAATTGACG |
| TCONS_00004045 | – | ATATTTGACGTTGTTTTATTTTTTTAAATAAAAGGACACTTATTGCGGCATAACTATAATAGTTAGACATATGCTATTGCAACATTTTTGTAAATAATAATGTGTTCTACAAAGTCGTAGTACATTATTTTATTCTATCATCAATAGTTTTCGCAGGGCACGCAATGTAAAGAATATTTTAGGTAATTATTGGAGTTTTAGTAAGGATCCCTAATTTTTTTCAAAAAAGATTATAGCCTATGTCACTCGGGAATAGTGTAGCTTCCAAACAGTGAAAGAATTTTTCAAATCGGTTCTGTAGTTCGGAGCCTATTTAATACAAACAAACAAACAAAACTTTCCTCTTTATAATATTAGTATAGAC |
| TCONS_00004112 | 5 | TATAAAAAAAATGTGTGCGTGTACTAGTGTACCACGTAAGAAGTGAAACTTGTTTATGGCCTTATTTTTCCGAAAAATGATCTACATGCAACTTTCTAGAAATTGGTTAAATAAAGTTAAATTAGATAAAGTTTAAACAAAAGGATTTCATTATCATAGACATGAATAAAAAAAAAATGTGTGCGTGTACTAGTGTACACACGTAAGAAGTGAAACTTGTTTATGGCCTTATTTTTCGAAAAATGATCTACAGTGCAACTTTCTAGAAATTGGTTAAATAAAGTTAAATTAGATAAAGTTTAAACAAAAGGATTTTATTATCATAGACATGAATACAAAAAAGTTAAATTAGATAAAGTTTAAACAAAAGGATTTTATTATCATAGACATGAATACAAAAAAGATGGCGCGTAACGGAAAAATGTGACGCGTAACCGAAAAATGTGACGGTAAATTTTTTTCCAACGCCGATAAGGAAGTTTCACTTCAAAAAATTAAAAAAAATTAAAAAAAAAGTAGCCTATGTTCATCAGGGACAATGTCGGCTTCTAATGGAAAAATAATTTTTCAAATCGGTCCAGTAGTTTCGGAGCCTATTCGAAACAAACAAACAAACAAATCTTTCCTCTTTATAATATTAGTATAGATATAGATAAATGAAAGTACGTCATACTTGTTGAACGTCAAAAGAACTACCGCCAATTCACAAGAATTAGCCTCCGTCCTGAGAAGAATTGGCAAGAAACTCAGCGGGCATGCCTTTTTTTTTTTAAATATTAGATTATTATTTTTTAATATTACATTATTTTTAAATATTCATTTTTACAATAAGTAATTATAACATAACAATTTTACAATATTGAAATGCCCGGAGCGAGCAACTCATTCCCACTTTGTGCAATCTTCTAGATAATCATTAACTTTATAGTAAGCTTTATTGCACGGATGTTCTGAACATCTTGTGGGATTTTGTTGTACAGGCGCACAGACAAACACCCGAATGAATTTCGTATCTTATGTAACCTACTCATCGCCACAACAAGCTTGTGTTTGTTCCTAGTATTTCTATTATGTATGTCCGACATTTTAGGAACTCCTCATATGTTTACGAACATTCATCAAATTTTCAAAGATGTAC |
| TCONS_00004113 | 5 | AAAAAAATGTGTGCGTGTACTAGTGTACCACGTAAGAAGTGAAACTTGTTTATGGCCTTATTTTTCCGAAAAATGATCTACATGCAACTTTCTAGAAATTGGTTAAATAAAGTTAAATTAGATAAAGTTTAAACAAAAGGATTTCATTATCATAGACATGAATACAAAAAAGATGGCGCGTAACGGAAAAATGTGACGCGTAACCGAAAAATGTGACGGTAAATTTTTTTCCAACGCCGATAAGGAAGTTTCACTTCAAAAAATTAAAAAAAATTAAAAAAAAAGTAGCCTATGTTCATCAGGGACAATGTCGGCTTCTAATGGAAAAATAATTTTTCAAATCGGTCCAGTAGTTTCGGAGCCTATTCGAAACAAACAAACAAACAAATCTTTCCTCTTTATAATATTAGTATAGATATAGATAAATGAAAGTACGTCATACTTGTTGAACGTCAAAAGAACTACCGCCAATTCACAAGAATTAGCCTCCGTCCTGAGAAGAATTGGCAAGAAACTCAGCGGGCATGCCTTTTTTTTTTTAAATATTAGATTATTATTTTTTAATATTACATTATTTTTAAATATTCATTTTTACAATAAGTAATTATAACATAACAATTTTACAATATTGAAATGCCCGGAGCGAGCAACTCATTCCCACTTTGTGCAATCTTCTAGATAATCATTAACTTTATAGTAAGCTTTATTGCACGGATGTTCTTTAATAGTTTTCTTAAACCTATGTATATGTAGGTTCTGAACATCTTGTGGGATTTTGTTGTACAGGCGCACAGACAAACACCCGAATGAATTTCGTATCTTATGTAACCTACTCATCGCCACAACAAGCTTGTGTTTGTTCCTAGTATTTCTATTATGTATGTCCGACATTTTAGGAACTCCTCATATGTTTACGAACATTCATCAAATTTTCAAAGATGTAC |
| TCONS_00004114 | 6 | GTACATCTTTGAAAATTTGATGAATGTTCGTAAACATATGAGGAGTTCCTAAAATGTCGGACATACATAATAGAAATACTAGGAACAAACACAAGCTTGTTGTGGCGATGAGTAGGTTACATAAGATACGAAATTCATTCGGGTGTTTGTCTGTGCGCCTGTACAACAAAATCCCACAAGATGTTCAGAACCTACATATACATAGGTTTAAGAAAACTATTAAAGAACATCCGTGCAATAAAGCTTACTATAAAGTTAATGATTATCTAGAAGATTGCACAAAGTGGGAATGAGTTGCTCGCTCCGGGCATTTCAATATTGTAAAATTGTTATGTTATAATTACTTATTGTAAAAATGAATATTTAAAAATAATGTAATATTAAAAAATAATAATCTAATATTTAAAAAAAAAAAGGCATGCCCGCTGAGTTTCTTGCCAATTCTTCTCAGGACGGAGGCTAATTCTTGTGAATTGGCGGTAGTTCTTTTGACGTTCAACAAGTATGACGTACTTTCATTTATCTATATCTATACTAATATTATAAAGAGGAAAGATTTGTTTGTTTGTTTGTTTCGAATAGGCTCCGAAACTACTGGACCGATTTGAAAAATTATTTTTCCATTAGAAGCCGACATTGTCCCTGATGAACATAGGCTACTTTTTTTTTAATTTTTTTTAATTTTTTGAAGTGAAACTTCCTTATCGGCGTTGGAAAAAAATTTACCGTCACATTTTTCGGTTACGCGTCACATTTTTCCGTTACGCGCCATCTTTTTTGTATTCATGTCTATGATAATGAAATCCTTTTGTTTAAACTTTATCTAATTTAACTTTATTTAACCAATTTCTAGAAAGTTGCATGTAGATCATTTTTCGGAAAAATAAGGCCATAAACAAGTTTCACTTCTTACGTGGTACACTAGTACACGCACACATTTTTTTTAT |
| TCONS_00004115 | 6 | GTACATCTTTGAAAATTTGATGAATGTTCGTAAACATATGAGGAGTTCCTAAAATGTCGGACATACATAATAGAAATACTAGGAACAAACACAAGCTTGTTGTGGCGATGAGTAGGTTACATAAGATACGAAATTCATTCGGGTGTTTGTCTGTGCGCCTGTACAACAAAATCCCACAAGATGTTCAGAACCTACATATACATAGGTTTAAGAAAACTATTAAAGAACATCCGTGCAATAAAGCTTACTATAAAGTTAATGATTATCTAGAAGATTGCACAAAGTGGGAATGAGTTGCTCGCTCCGGGCATTTCAATATTGTAAAATTGTTATGTTATAATTACTTATTGTAAAAATGAATATTTAAAAATAATGTAATATTAAAAAATAATAATCTAATATTTAAAAAAAAAAAGGCATGCCCGCTGAGTTTCTTGCCAATTCTTCTCAGGACGGAGGCTAATTCTTGTGAATTGGCGGTAGTTCTTTTGACGTTCAACAAGTATGACGTACTTTCATTTATCTATATCTATACTAATATTATAAAGAGGAAAGATTTGTTTGTTTGTTTGTTTCGAATAGGCTCCGAAACTACTGGACCGATTTGAAAAATTATTTTTCCATTAGAAGCCGACATTGTCCCTGATGAACATAGGCTACTTTTTTTTTAATTTTTTTTAATTTTTTGAAGTGAAACTTCCTTATCGGCGTTGGAAAAAAATTTACCGTCACATTTTTCGGTTACGCGTCACATTTTTCCGTTACGCGCCTATGATAATAAAATCCTTTTGTTTAAACTTTATCTAATTTAACTTTATTTAACCAATTTCTAGAAAGTTGCACTGTAGATCATTTTTCGAAAAATAAGGCCATAAACAAGTTTCACTTCTTACGTGTGTACACTAGTACACGCACACATTTTTTTTTTATTCATGTCTATGATAATGAAATCCTTTTGTTTAAACTTTATCTAATTTAACTTTATTTAACCAATTTCTAGAAAGTTGCATGTAGATCATTTTTCGGAAAAATAAGGCCATAAACAAGTTTCACTTCTTACGTGGTACACTAGTACACGCACACATTTTTTTTAT |
| TCONS_00004248 | – | TTTTGCGGGTTTGGTTTTTATTACACGATTAGCTGACCCGGCAGACTTCGTAGTGCCTCAATCGATAAATAAAAGACCTAAACTTTTGTATAAAATAAACTTAAAACAAACAAAAGGAATCCGTCCGACGGGGGACACATCAAAGGTAAAACAAAATTGTTATTTTTATTTAATTCTGGGCATTTTCATATTTATCTACCTTTTAAACCTTCTCTGGACTTCCACAAATAATTCAAGACCAAAATTAGCCAAATCGGTCCAGCCGTTCTCGAGTTTTAGCGAGACTAACGAACAGCAATTCATTTTTATATATATAGATAGAAGAAGAAGATT |

Note: Transcripts sharing the same Locus number show high sequence similarity and most likely represent alternative isoforms or redundant transcript assemblies originating from the same genomic locus rather than independent lncRNAs; “–” denotes a transcript with no near-identical counterpart in this table.

**Predicted milRNAs in *Nosema bombycis***

| milRNA | Mature milRNA | Precursor milRNA |
| --- | --- | --- |
| milRNA-13 | uaaagacggaaauuaguaagcgcua | uaaagacggaaauuaguaagcgcuauuuuguuaaaauuugcuucuccauaguuucaguuuuc |
| milRNA-14 | ugaggauuguucuguaggcauaguc | uuauuucccuguucaauucugaagucuccgcagaucuuuugaggauuguucuguaggcauaguc |
| milRNA-15 | ugcgcgucuauaaauuuugaaaagc | uguuucugaaauuuccuaugccgguuuauauugggaaagcgcccuuuuugcaaacgauaugggcccaugcgcgucuauaaauuuugaaaagc |
| milRNA-16 | uuuaaggcgugguaucagauuugc | uuuaaggcgugguaucagauuugcaagcuauaugucugccuguuucgaacuuuuga |
| milRNA-18 | uagaacguaucucugacuuugucc | uaaauuuuuaaagacuaccuuucucaaaaaaaaaauagaacguaucucugacuuugucc |
| milRNA-19 | uuuuagcguccgaauguauaguuuc | ucuuugucaguaaauguugaaagaauaaaugccucauuaaacuuuaucauaccuauuuuagcguccgaauguauaguuuc |
| milRNA-20 | uaugguccucuucagaauuugaau | uaugguccucuucagaauuugaauauaagauuauauugucuagauaagcaagaacaaauuuuucucuuaaaccugcaaggaccugaucc |
| milRNA-21 | ucauaauaggagcugcaaagacgu | aaaguugggcuuuacuaugaagaugucauaauaggagcugcaaagacgu |
| milRNA-22 | uggaaaaggacuuuuuaagggaau | uggaaaaggacuuuuuaagggaauauuuuuauaaaaauaaaaaaaaauucucucaucacuuuauccaauacac |
| milRNA-23 | uggaucaguaaccaucagcggau | uggaucaguaaccaucagcggaucaggagaaucacgggcgauuugcugaugaucgguagcugaucccgggu |
| milRNA-24 | ucggaggcuuaaauuccaugacguu | ucggaggcuuaaauuccaugacguuaauguguuuuuauuaaggucauuuucuugagccacauccugacuuu |
| milRNA-25 | uuuccgauauuuugggcguaaacc | uuuccgauauuuugggcguaaaccguaguauuaugagggacaaaaaauuguaacuaugauuuacuuaaagauguucaaaaauuggaucuu |
