# Supplementary material for: Cross-kingdom noncoding RNA regulation facilitates Nosema bombycis proliferation
Source: Eng Microbiol. 2026 Jun 3;6(3):100278. doi: 10.1016/j.engmic.2026.100278 (PMC13276322; doi:10.1016/j.engmic.2026.100278)
Supplement: Supplementary file 3 [file mmc3.docx]

**Table S3 Primers used in this study**

| Name | Primer information | | | | | | Sequence（5'-3'） | |  |
| --- | --- | --- | --- | --- | --- | --- | --- | --- | --- |
| TCONS_00000152 | qPCR-F | | | | | | ATTTGCATGCCTTGAAGCCG | |  |
|  | qPCR-R | | | | | | AGACTCCCTATCGGCACAGT | |  |
| TCONS_00000968 | qPCR-F | | | | | | ATAGTGGTGCATGGCCGTTT | |  |
|  | qPCR-R | | | | | | GCGCGTGCAACCCAGATTAT | |  |
| TCONS_00002970 | qPCR-F | | | | | | AAATCGGAGGGCAAATCGAG | |  |
|  | qPCR-R | | | | | | CGCTTCTGTTCATCCAGTTAGG | |  |
| TCONS_00003738 | qPCR-F | | | | | | TGGCGAAAGACCAATCGAAC | |  |
|  | qPCR-R | | | | | | TTCTTGCCCACTTGAGTATCG | |  |
| TCONS_00001630 | qPCR-F | | | | | | AAATCGGAGGGCAAATCGAG | |  |
|  | qPCR-R | | | | | | CGCTTCTGTTCATCCAGTTAGG | |  |
| TCONS_00002816 | qPCR-F | | | | | | GGTCGATCTCTTCCCCCTCT | |  |
|  | qPCR-R | | | | | | CTTAACACCAGGTGGGCTGT | |  |
| TCONS_00004045 | qPCR-F | | | | | | AATAGTTTTCGCAGGGCACG | |  |
|  | qPCR-R | | | | | | GCTCCGAACTACAGAACCGA | |  |
| TCONS_00002522 | qPCR-F | | | | | | GGAACGAAGTTCCCTAACCG | |  |
|  | qPCR-R | | | | | | GAGGTTTCGCAACAACCCAC | |  |
| TCONS_00004248 | qPCR-F | | | | | | GCGGGTTTGGTTTTTATTACACG | |  |
|  | qPCR-R | | | | | | AAACTCGAGAACGGCTGGAC | |  |
| TCONS_00000141 | qPCR-F | | | | | | GTCCAGAACACAAAGAGACGAC | |  |
|  | qPCR-R | | | | | | TTCGGTGCCTTTTTCGGATC | |  |
| TCONS_00004073 | qPCR-F | | | | | | AGACTTCGTAGTGCCTCAATCG | |  |
|  | qPCR-R | | | | | | TAAAACTCGAGAACGGCTGGAC | |  |
| TCONS_00000007 | qPCR-F | | | | | | ACCAATCAGAGCTACCGACAAC | |  |
|  | qPCR-R | | | | | | ATCTTGTTCTGCTCTGGAGACC | |  |
| TCONS_00000008 | qPCR-F | | | | | | TGCCATGGGATCAATAGGATGTC | |  |
|  | qPCR-R | | | | | | TGGGGCGCGAACCTAAAAAG | |  |
| TCONS_00002695 | qPCR-F | | | | | | TCGGATGCACGGTTCAGTAG | |  |
|  | qPCR-R | | | | | | TCTCACCCCCACAAAGATTCTC | |  |
| TCONS_00003941 | qPCR-F | | | | | | AAAAAGATGGCGCGTAACGG | |  |
|  | qPCR-R | | | | | | AGCCGACATTGTCCCTGATG | |  |
| TCONS_00002914 | qPCR-F | | | | | | TCCACTTGTGCTGACCAATC | |  |
|  | qPCR-R | | | | | | AGATGGTTGATGGATTCCTTAGC | |  |
| *NbPTP2* | qPCR-F | | | | | | ATCAGAACAATGGCATCAGTA | |  |
|  | qPCR-R | | | | | | ACATTCGTTAATAAGTGGGG | |  |
| *NbEIF2α* | qPCR-F | | | | | | AGGACGAGTTTGACATTT | |  |
|  | qPCR-R | | | | | | TCCGCTATTTTAGGAGTA | |  |
| *NbTCP1* | qPCR-F | | | | | | TGAACACGAGAAAATGAAGGCA | |  |
|  | qPCR-R | | | | | | TCCGCGTGTCTATTGGTTGT | |  |
| *NbHSP70* | qPCR-F | | | | | | TGATTCTGTTGGGATTGC | |  |
|  | qPCR-R | | | | | | TTCTGAAGTAGCGTATTG | |  |
| *NbSWP5* | qPCR-F | | | | | | ATGAAAGAAAATAAGAATGTGCCG | |  |
|  | qPCR-R | | | | | | CGGTTATTTATCCGAAGGTG | |  |
| *NbssurRNA* | qPCR-F | | | | | | ACGGAAGAATACCACAAGGAGT | |  |
|  | qPCR-R | | | | | | CACTACATCTGTCTAAATGAGGGTC | |  |
| *Nbβ-Tubulin* | qPCR-F | | | | | | CTTTGGACAATCTGGTGCTG | |  |
|  | qPCR-R | | | | | | GAGAAGGGTTCCCATTCCTG | |  |
| milRNA-13 | poly(A) tailed forward | | | | | | AAGCGACCTAAAGACGGAAATTAGT | |  |
| milRNA-14 | poly(A) tailed forward | | | | | | AACAAGTGAGGATTGTTCTGTAGGC | |  |
| milRNA-15 | poly(A) tailed forward | | | | | | AACACGCTGCGCGTCTATAAATTT | |  |
| milRNA-16 | poly(A) tailed forward | | | | | | AACAAGTTTAAGGCGTGGTATCAGA | |  |
| milRNA-18 | poly(A) tailed forward | | | | | | AACACGCTAGAACGTATCTCTGACT | |  |
| milRNA-19 | poly(A) tailed forward | | | | | | AACACGCTTTTAGCGTCCGAAT | |  |
| milRNA-20 | poly(A) tailed forward | | | | | | AACACGCTATGGTCCTCTTCAGAAT | |  |
| milRNA-21 | poly(A) tailed forward | | | | | | AACACGCTCATAATAGGAGCTGC | |  |
| milRNA-22 | poly(A) tailed forward | | | | | | AACGGCTGGAAAAGGACTTTTTAAG | |  |
| milRNA-23 | poly(A) tailed forward | | | | | | AACAAGTGGATCAGTAACCATCAGC | |  |
| milRNA-24 | poly(A) tailed forward | | | | | | AACAAGTCGGAGGCTTAAATTCCAT | |  |
| UPM | RACE | | | | | | CTAATACGACTCACTATAGGGCAAGCAGTGGTATCAACGCAGAGT | |  |
| UPM short | RACE | | | | | | CTAATACGACTCACTATAGGGC | |  |
| TCONS_00002914 | 2914-5’GSP-1 | | | | | | TGTCTCTCTCTGAGTTCCTTGGGGT | |  |
|  | 2914-3’GSP-1 | | | | | | GGGGAAAGGACAGCCAGAGAGAGCGCGC | |  |
|  | 2914-3’GSP-2 | | | | | | TCACTTAACAACCCCCCCTCCACCATGT | |  |
|  | 2914(EcoRI)-F | | | | | | CGGAATTCAGGTGCTCTGGTGTGTTTA | |  |
|  | 2914(NotI)-R | | | | | | AAGGAAAAAAGCGGCCGCTGTTGGTAATTACTTTTATTTTCT | |  |
|  | 2914(Xba I)-F | | | | | | TGCTCTAGAAGGTGCTCTGGTGTGTTTA | |  |
|  | 2914(Fse I)-R | | | | | | GGCCGGCCTGTTGGTAATTACTTTTATTTTCT | |  |
| bmo-bantam-5p | | qPCR-F | | | | | | GCCGCCTGGTTTTCATAATGATTTGAC |  |
| bmo-miR-2770-3p | | qPCR-F | | | | | | GGCGGCTTATCCCCGTGTACTGTTAG |  |
| bmo-miR-2808a-3p | | qPCR-F | | | | | | AACAAGCGGTGGTAGATTCTGC |  |
| bmo-miR-2808c | | qPCR-F | | | | | | GGCGTGGTGGTAGATTCAGCGAAACA |  |
| bmo-miR-3206 | | qPCR-F | | | | | | AACACGCTATCCATCAGTTTGTAGG |  |
| bmo-miR-3247 | | qPCR-F | | | | | | GGCGTTAAATTGTAAAAAAGCGTGGG |  |
| milRNA-1 | | qPCR-F | | | | | | GGGCTAAGACGTCCGGTGCATTCG |  |
| bmo-miR-2808a-3p | | mi2808(Asc I)-R | | | | | | GGCGCGCCAAAAAACGTACTTCGCAGAATCTACCACCGGTAGAGCACGATATTTT |  |
| U6 Promoter | | miRNA-u6(Bgl II)-F | | | | | | AGATCTAGGTTATGTAGTACACATTGT |  |
| bmo-miR-2808a-3p inhibitor | | | | | F | | GCUACUUCGCAGAAUCUACCACCG | | |
|  |  |  |  |  | R | | CGGUGGUAGAUUCUGCGAAGUACG | | |
| *NBO_7g0065* | | | | | qPCR-F | | GAGATTTATGCCCGAAAAGGAC | | |
|  |  |  |  |  | qPCR-R | | GCCATTGCTCTTTCAGTTACTT | | |
| *NBO_508g0015* | | | | | qPCR-F | | GTGAACGCTAAACAACTGAGTT | | |
|  |  |  |  |  | qPCR-R | | GCTCTTAATCGTTTCATGGCAT | | |
| *NBO_53g0029* | | | | | qPCR-F | | GCTTTGGCTAAATTGCATTGTG | | |
|  |  |  |  |  | qPCR-R | | GAAGGTTTTGTAGAATCAACTGGAC | | |
| *NBO_6g0087* | | | | | qPCR-F | | GGTTTGGTTGTGTCATTAACGA | | |
|  |  |  |  |  | qPCR-R | | TTTCAGACTGTAACCGAGAGAC | | |
| *NBO_58g0005* | | | | | qPCR-F | | ATTTTGATAGCACCATGGTGTC | | |
|  |  |  |  |  | qPCR-R | | ATTTGGCTCCTGTTGACACTAT | | |
| *NBO_33g0018* | | | | | qPCR-F | | ACTCGACTTAGGAACGACATTT | | |
|  |  |  |  |  | qPCR-R | | CCAAATAGCGTTGTAGTAAGCC | | |
| *NBO_464g0004* | | | | | qPCR-F | | CAGTTCATACACAAGAACAAGC | | |
|  |  |  |  |  | qPCR-R | | CTCATCAATCTTGTTATCGTCTGG | | |
| *NBO_10g0112* | | | | | qPCR-F | | GAACAACAAATCGTACCACCAA | | |
|  |  |  |  |  | qPCR-R | | GCCACTTTTCCCATGAATACTC | | |
| *NBO_427g0006* | | | | | qPCR-F | | GGCTCTTTCTAAAGAATCCCCT | | |
|  |  |  |  |  | qPCR-R | | GCTAGGGTAACTTCTAGTAAATCAC | | |
| mi2808-NBO_58g0005 | | | | | F | | CTAGAACAGTCACAGGGACTTACCACTCCCGG | | |
|  |  |  |  |  | R | | GAGTGGTAAGTCCCTGTGACTGTT | | |
| mi2808-NBO_58g0005-mut | | | | | F | | CTAGAACAGTCACAGGGACTATGGTGGCCCGG | | |
|  |  |  |  |  | R | | GCCACCATAGTCCCTGTGACTGTT | | |
| siRNA-1 | | | | F | | GGCTCTTTCTAAAGAATCCCCT | | | |
|  |  |  |  | R | | GCTAGGGTAACTTCTAGTAAATCAC | | | |
| siRNA-2 | | | | F | | CTAGAACAGTCACAGGGACTTACCACTCCCGG | | | |
|  |  |  |  | R | | GAGTGGTAAGTCCCTGTGACTGTT | | | |
| siRNA-3 | | | | F | | CTAGAACAGTCACAGGGACTATGGTGGCCCGG | | | |
|  |  |  |  | R | | GCCACCATAGTCCCTGTGACTGTT | | | |
