# Supplementary material for: Cross-kingdom noncoding RNA regulation facilitates Nosema bombycis proliferation
Source: Eng Microbiol. 2026 Jun 3;6(3):100278. doi: 10.1016/j.engmic.2026.100278 (PMC13276322; doi:10.1016/j.engmic.2026.100278)
Supplement: Supplementary file 4 [file mmc4.docx]

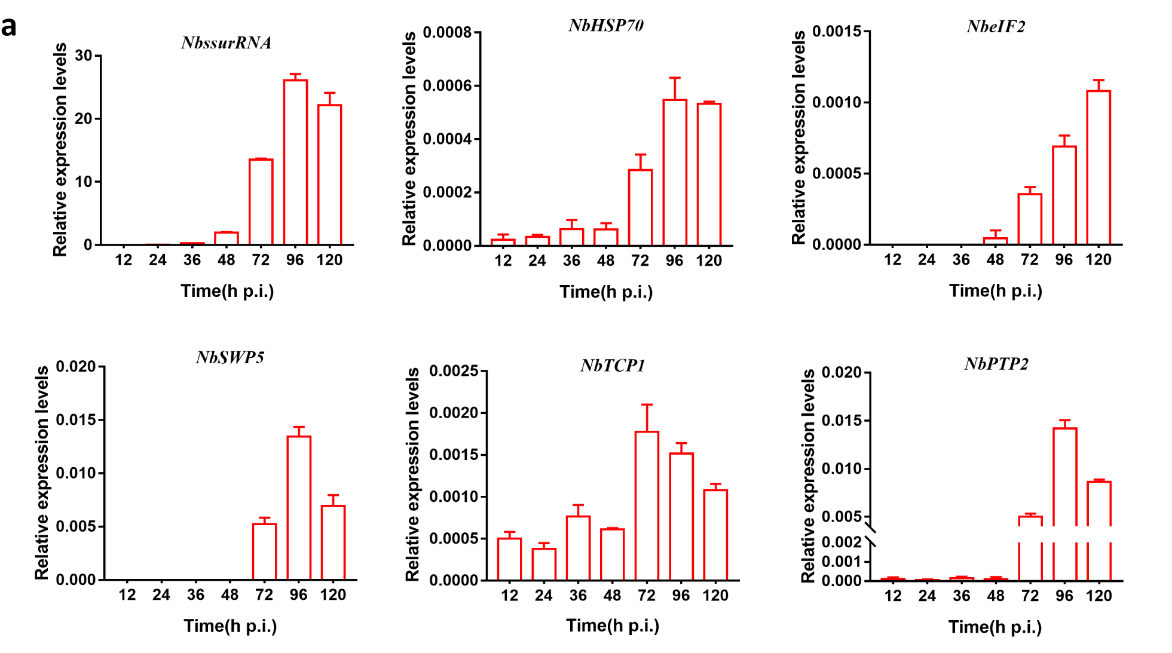


**S1 Fig. Expression of *N. bombycis* genes at different time points post-infection in the midgut of *B. mori*.**

Expression of *NbssurRNA*, *NbHSP70*, *NbeIF2*, *NbSWP5*, *NbTCP1*, and *NbPTP2* in the midguts of silkworms infected with *N. bombycis* at different time points.


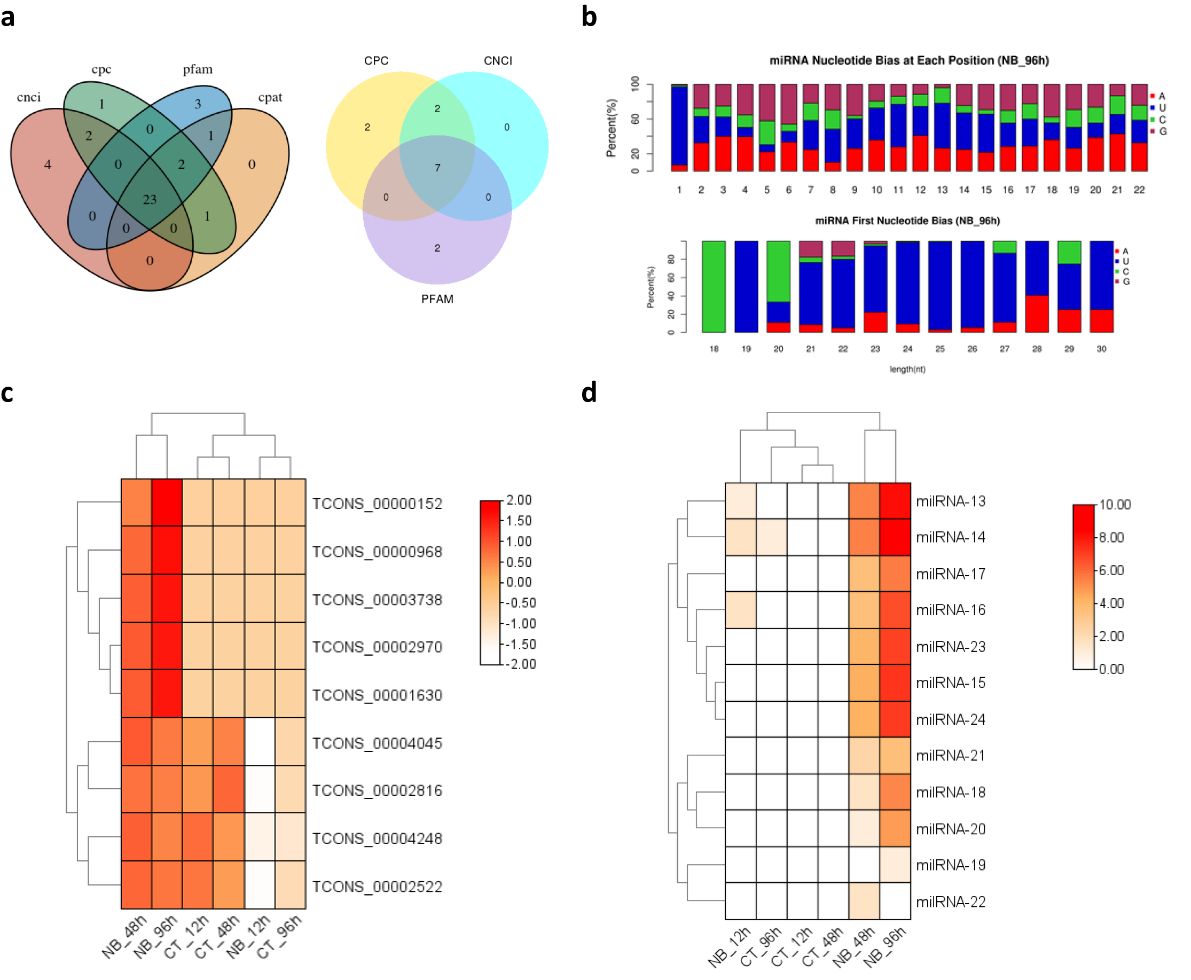


**S2 Fig. Expression profile analysis of milRNAs and lncRNAs in *N. bombycis***

(a) Identification of novel lncRNAs in cell and midgut samples using four coding-potential prediction tools (CPC, CNCI, CPAT, and Pfam), and only the intersecting candidates were retained as novel lncRNAs. (b) Nucleotide composition bias of novel milRNAs, including nucleotide preference at each position and first-nucleotide bias among milRNAs of different lengths. (c) Cluster heatmap analysis of differentially expressed LNCRNAs of *N. bombycis* in midgut samples. (d) Cluster heatmap analysis of differentially expressed milRNAs of *N. bombycis*.


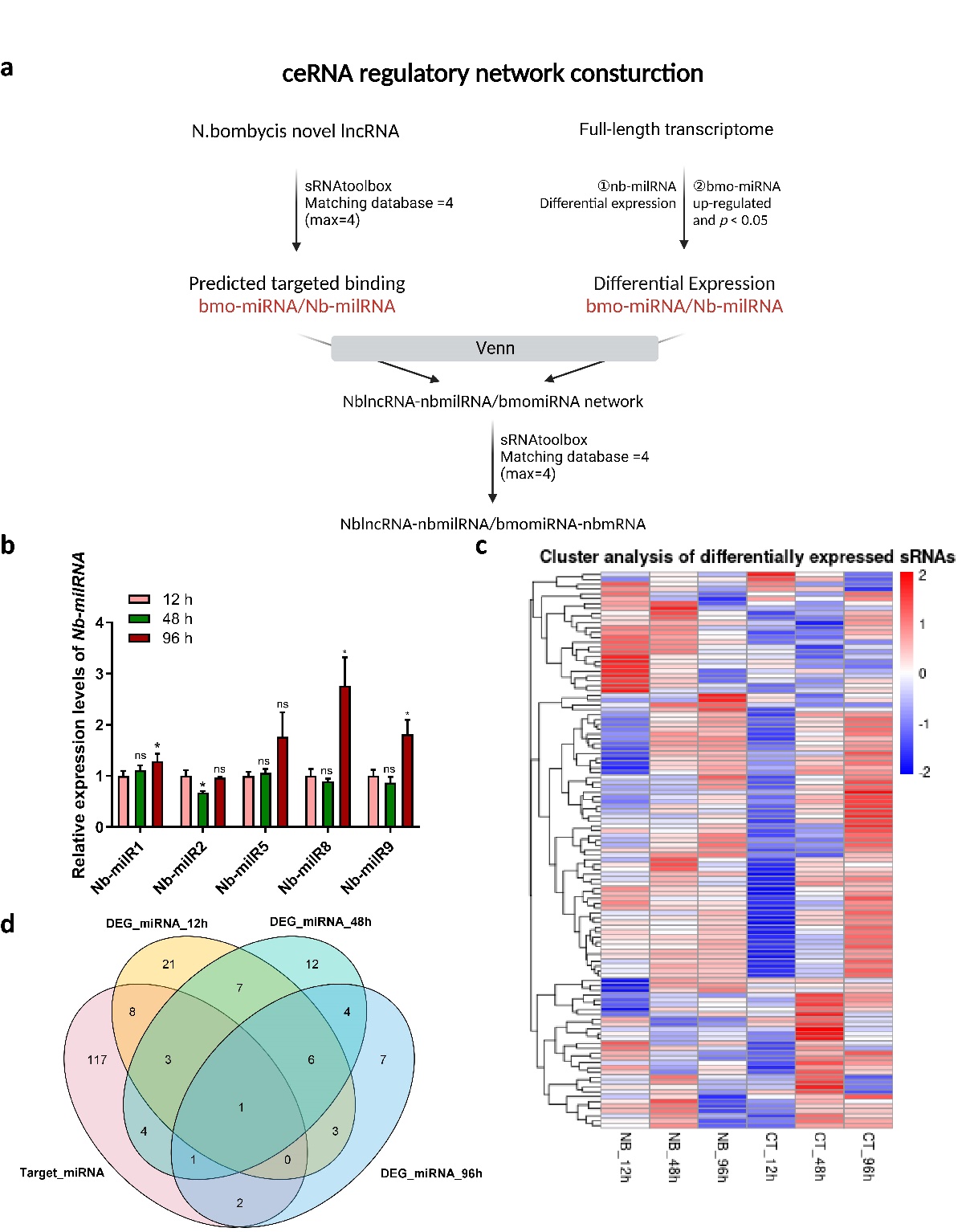


**S3 Fig. Construction of NblncRNA–NbmiRNA/BmmiRNA–NbmRNA regulatory network**

(a) Schematic overview of the strategy used to construct the ceRNA regulatory network in *N. bombycis* based on predicted lncRNA–miRNA interactions and differential expression analysis. (b) Differential expression of target NbmilRNAs. (c) Cluster heatmap analysis of differentially expressed miRNAs in silkworms after *N. bombycis* infection. (d) Venn diagram of NblncRNA-targeted miRNAs and significantly upregulated silkworm miRNAs at different time points after *N. bombycis* infection.**
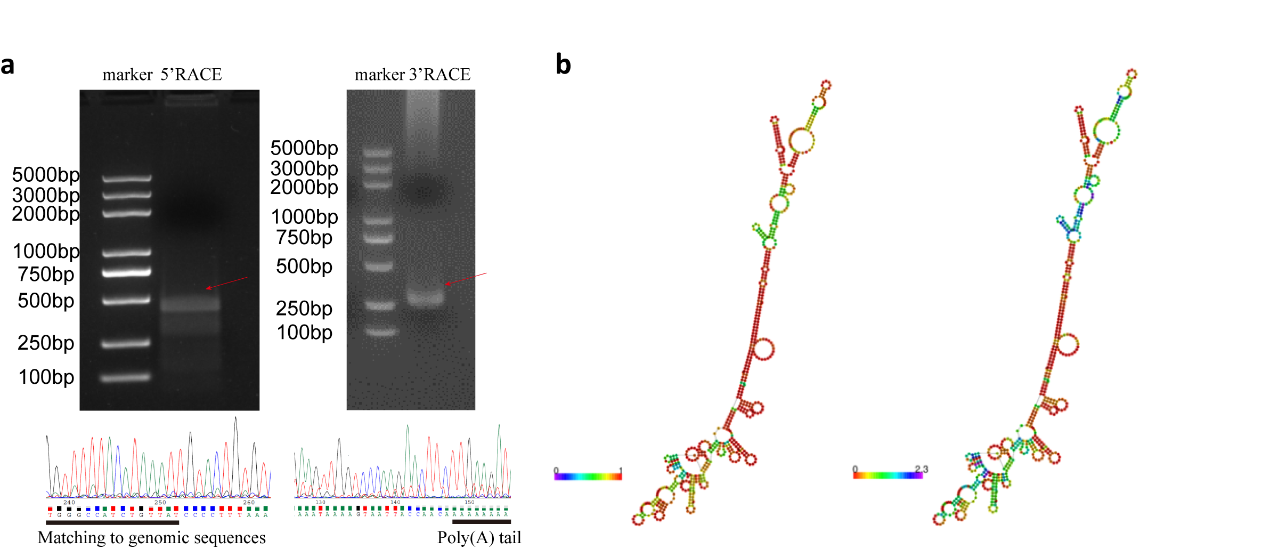
**

**S4 Fig. Full-length cloning and secondary structure prediction of *NbLNC2914***

(a) 5’ RACE and 3’ RACE amplification of *NbLNC2914*. (b) Minimum free energy structure (left) and centroid secondary structure (right) of *NbLNC2914*.

**
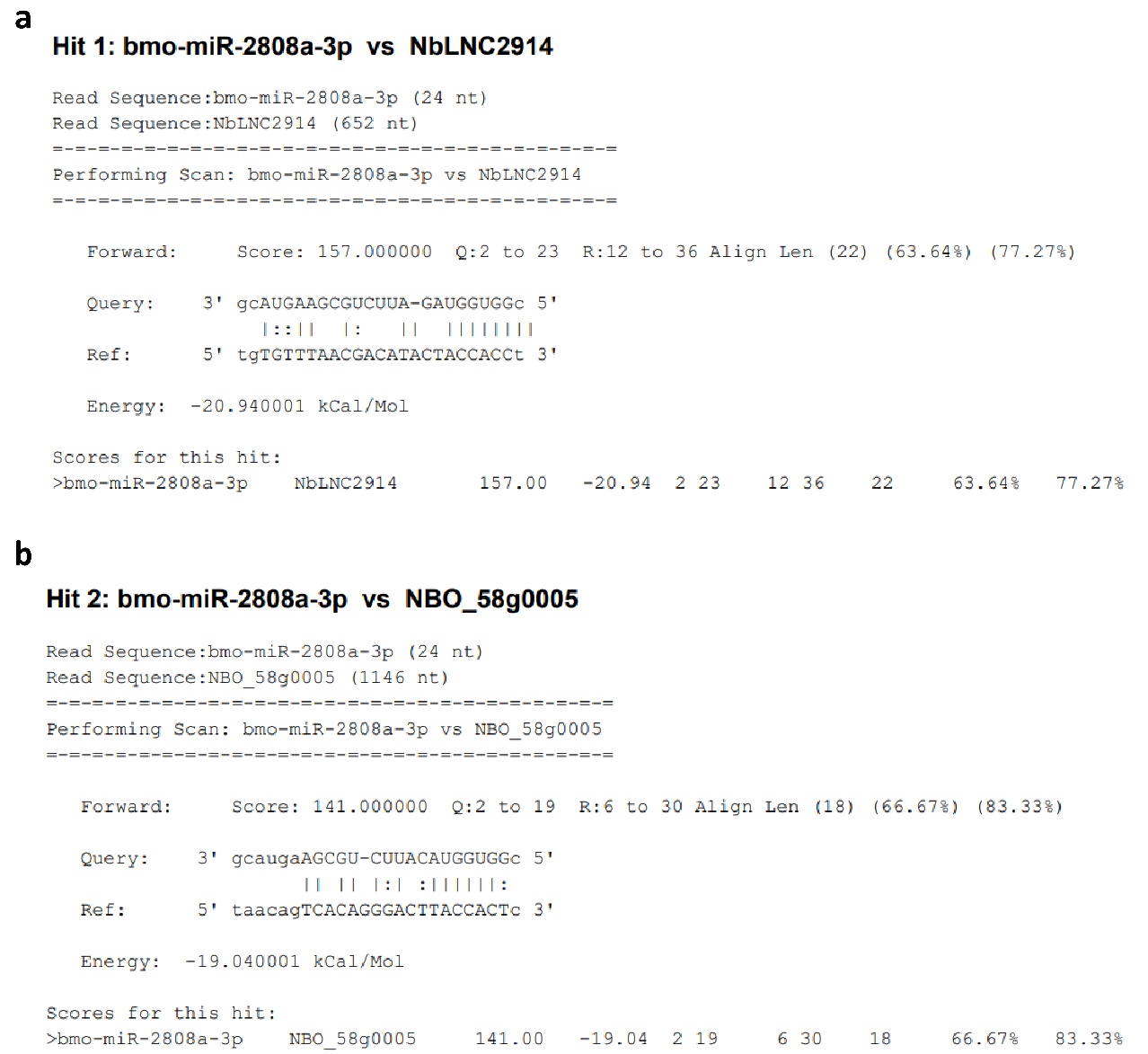
**

**S5 Fig. miRanda-predicted interactions between bmo-miR-2808a-3p and its target transcripts.**

(a) miRanda prediction of the interaction between bmo-miR-2808a-3p and NbLNC2914, showing a score of 157.00 and a minimum free energy of -20.94 kcal/mol. (b) miRanda prediction of the interaction between bmo-miR-2808a-3p and *NBO_58g0005*, showing a score of 141.00 and a minimum free energy of -19.04 kcal/mol.
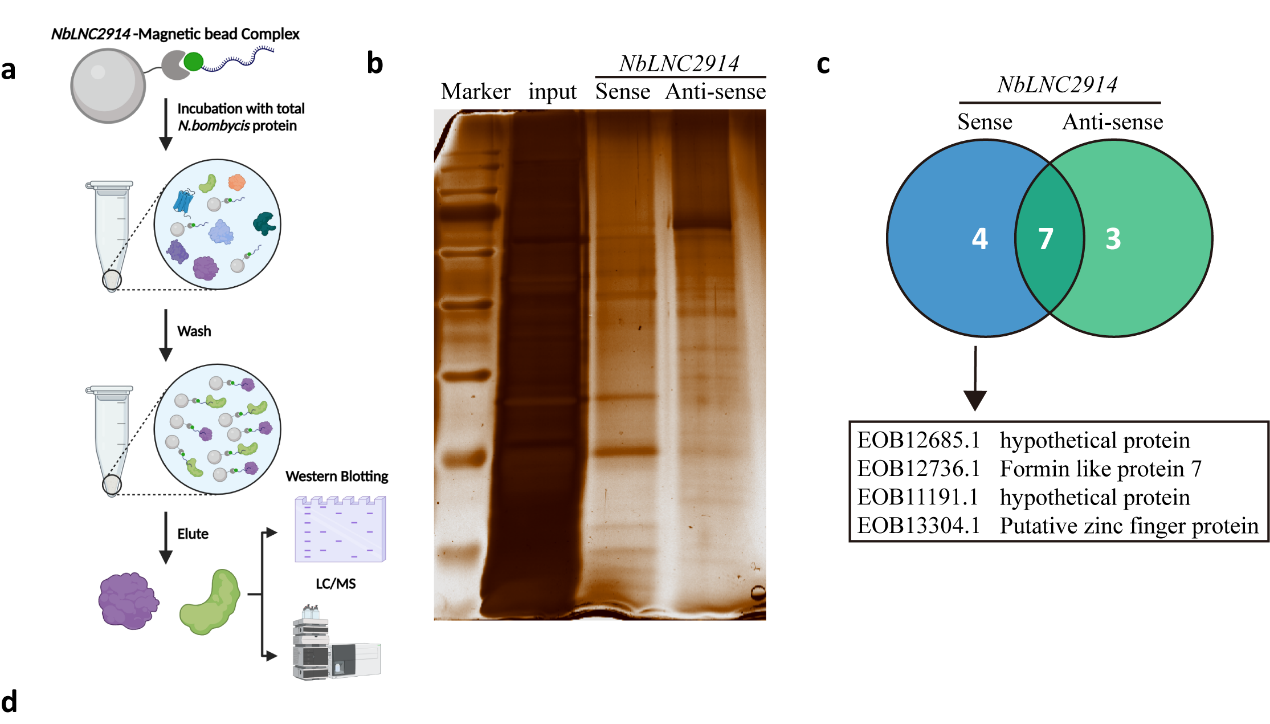


**S6 Fig. Identification of *NbLNC2914*-binding proteins in *N. bombycis***

(a) Schematic of the pull-down assay using *NbLNC2914* magnetic bead complexes incubated with total *N. bombycis* protein, followed by washing, elution, and analysis by Western blotting and LC/MS. (b) SDS-PAGE analysis of proteins bound to sense and anti-sense *NbLNC2914*. (c) Venn diagram showing the number of proteins specifically interacting with sense or anti-sense *NbLNC2914* and list of overlapping candidate proteins identified by LC/MS.
